# Unexpectedly High Levels of Cryptic Diversity Uncovered by a Complete DNA Barcoding of Reptiles of the Socotra Archipelago

**DOI:** 10.1371/journal.pone.0149985

**Published:** 2016-03-01

**Authors:** Raquel Vasconcelos, Santiago Montero-Mendieta, Marc Simó-Riudalbas, Roberto Sindaco, Xavier Santos, Mauro Fasola, Gustavo Llorente, Edoardo Razzetti, Salvador Carranza

**Affiliations:** 1 CIBIO, Centro de Investigação em Biodiversidade e Recursos Genéticos, InBIO Laboratório Associado, Universidade do Porto, Vairão, Portugal; 2 Institute of Evolutionary Biology (CSIC-UPF, Consejo Superior de Investigaciones Científicas- Universitat Pompeu Fabra), Barcelona, Spain; 3 Departament de Biologia Animal, Facultat de Biologia, Universitat de Barcelona, Barcelona, Spain; 4 Museo Civico di Storia Naturale, Carmagnola (TO), Italy; 5 Dipartimento Scienze della Terra e dell’Ambiente, Università degli studi di Pavia, Pavia, Italy; 6 Museo di Storia Naturale, Università degli studi di Pavia, Pavia, Italy; State Natural History Museum, GERMANY

## Abstract

Few DNA barcoding studies of squamate reptiles have been conducted. Due to the significance of the Socotra Archipelago (a UNESCO Natural World Heritage site and a biodiversity hotspot) and the conservation interest of its reptile fauna (94% endemics), we performed the most comprehensive DNA barcoding study on an island group to date to test its applicability to specimen identification and species discovery. Reptiles constitute Socotra’s most important vertebrate fauna, yet their taxonomy remains under-studied. We successfully DNA-barcoded 380 individuals of all 31 presently recognized species. The specimen identification success rate is moderate to high, and almost all species presented local barcoding gaps. The unexpected high levels of intra-specific variability found within some species suggest cryptic diversity. Species richness may be under-estimated by 13.8–54.4%. This has implications in the species’ ranges and conservation status that should be considered for conservation planning. Other phylogenetic studies using mitochondrial and nuclear markers are congruent with our results. We conclude that, despite its reduced length (663 base pairs), cytochrome c oxidase 1, COI, is very useful for specimen identification and for detecting intra-specific diversity, and has a good phylogenetic signal. We recommend DNA barcoding to be applied to other biodiversity hotspots for quickly and cost-efficiently flagging species discovery, preferentially incorporated into an integrative taxonomic framework.

## Introduction

The accuracy of delimiting species is fundamental in specimen identification and species discovery. Over a decade ago, DNA barcoding was proposed as a fast, cost-efficient and simple taxonomic method based on the use of a unique, short and standardized gene region (cytochrome c oxidase 1, COI, for animals) for identifying specimens and expediting discovery of putative new species [1]. A crucial premise of DNA barcoding is that genetic variation within species (intra-specific) is lower than among species (inter-specific) [1–3], i.e., that a ‘barcoding gap’ exists [4] which allows unknown specimens to be identified as an existing species or flagged as a putative new species. While some previous studies have confirmed the presence of a global barcoding gap, such as in birds [3], fish [5] or butterflies [6], others have concluded that it does not always exist, sometimes disregarding the importance of local barcoding gaps (i.e., a query sequence being closer to a conspecific than a different species) [7]. The accuracy of species delimitation also depends on the completeness of the DNA reference library, the geographic extent of sampling, the intensity of intra-specific sampling, and the divergence time among closely-related species [8–10].

Due to technical problems regarding the amplification of COI sequences, herpetologists have mainly been using the 16S rRNA gene instead [11], and few DNA barcoding studies on non-avian reptiles have been conducted so far, with still fewer on islands [12]. Consequently, there is a lack of DNA barcodes for reptiles even though they are one of the best models for evolutionary, biogeographic, and phylogeographic studies, and are known to present high levels of cryptic diversity [13,14]. Recent advances in primer development have facilitated DNA barcoding of reptiles and the launch of a global initiative, ‘Cold Code’, aimed at barcoding all herpetofauna [15].

As DNA barcoding has proven to be an invaluable tool for specimen identification and preliminary species discovery for many taxa, it can greatly reduce problems that arise from morphological taxonomy approaches, while facilitating biologically-sound conservation planning [16]. This is especially important in under-sampled, and biodiversity hotspot areas such as islands.

**Fig 1:**
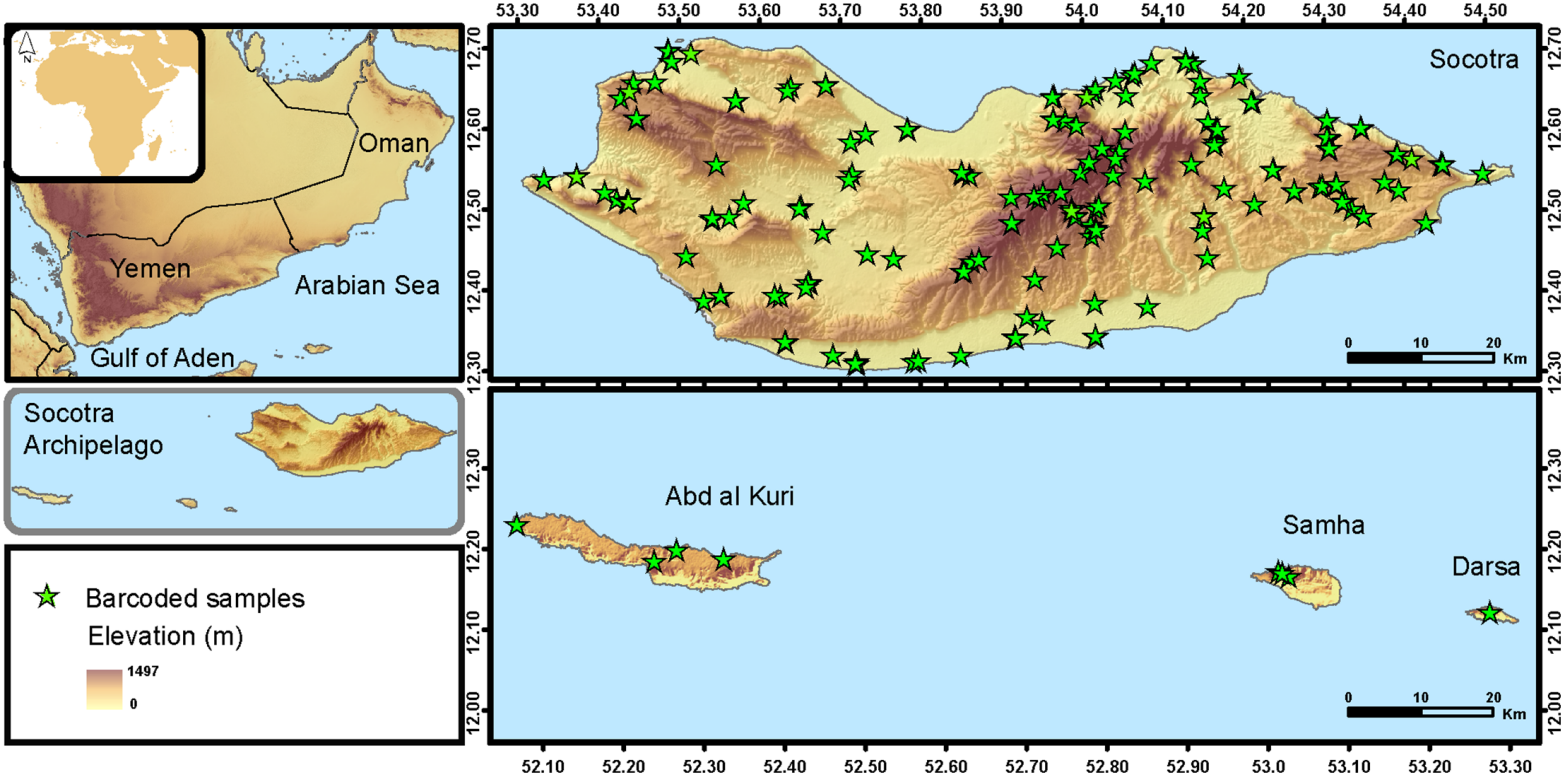
Sampling site and localities. Map showing the geographic situation of the Socotra Archipelago and the sampling localities for all the 380 barcoded individuals included in this study. Maps were drawn using DIVA-GIS v.7.5 (available at http://www.diva-gis.org; digital elevation model freely available at http://earthobservatory.nasa.gov/).

Socotra (a governorate currently belonging to Yemen) is considered one of the most difficult-access and distinct archipelagos in the world [17]. It is comprised of four islands of continental origin (Fig 1); in brief a block of pre-Cambrian Gondwanaland located in southern Oman, was separated from continental Arabia starting around 20 million years ago with the opening of the Gulf of Aden [18]. Presently Socotra lies in the northwest Indian Ocean approximately 100 km from the Horn of Africa [19].

The complex geological history of the Socotra Archipelago, with a long period of isolation from the mainland, together with its topography, the presence of many different microclimates and habitats, and centuries of sustainable traditional management, are considered the main causes of the origin and persistence of its high levels of endemic species and genera [20,21]. For example, 37% of its 825 plant species and 95% of its more than 100 land snail species are endemic [22]. However, threats to its biodiversity related to overgrazing, the introduction of exotic species, unsustainable exploitation of resources, and infrastructure and tourism development have been increasing since the last decade [17]. Due to its high number of endemic and threatened species, Socotra was included as a Horn of Africa biodiversity hotspot, which is one of the most threatened in the world [23], and was designated a UNESCO Natural World Heritage site in 2008.

With only one endemic mammal, 6 endemic bird species and no amphibians, reptiles constitute the most relevant Socotran vertebrate fauna with 31 species, many of which may have arisen from adaptive radiation [24]. If one excludes the two recently introduced species, *Hemidactylus robustus* and *Hemidactylus flaviviridis*, all native species are endemic [25–27]. There is a very high level of endemism at both species (29 of 31, 94%) and genus levels (5 of 12, 42%). At the species level, endemicity may be even higher, as preliminary phylogenetic studies have uncovered substantial hidden diversity [24,28]. Socotran reptiles also constitute a keystone group in the trophic chain, both as insect predators [29] and as prey for birds [30]. Moreover, some species have strict associations with specific habitats [27]. In this regard, the construction of a DNA-based reference library for all the reptiles of the Socotra Archipelago can serve as an integrative and useful tool for monitoring Socotran biodiversity.

To our knowledge, this work represents one of the most comprehensive DNA barcoding studies on islands. It includes a complete class sampling and an optimized site sampling strategy designed to evaluate the DNA barcoding performance for both specimen identification and species discovery. The specific aims were to: i) generate a DNA reference barcode library for the reptiles of the Socotra Archipelago, ii) test the effectiveness of the library for future specimen identification purposes using different distance-based and tree-based techniques, iii) explore previously unrecognized diversity by applying species delimitation methods, and iv) test the robustness of phylogenetic inference and species delimitation using COI compared with previous marker studies.

## Materials and Methods

### Ethics statements

No in vivo experiments were performed. Animals were just measured, sexed, DNA-sampled (1 mm of tail tips, clipped by hand and collected by authors) and then released at the capture site. Caudal autotomy is natural in most reptiles and clipped tails regenerate soon after. There is no Institutional Animal Care and Use Committee (IACUC) or ethics committee in neither CIBIO-InBIO nor in Yemen/Socotra. Licenses were provided by the national and regional committees for scientific sampling of biologic tissues, the Council of Minister of the Environmental Protection Authority of Yemen, Socotra branch, to perform fieldwork in Socotra that approved all sampling procedures in all species (threatened and non-threatened). All animals were put in cloth bags during sampling to ameliorate stress and no animals were harmed. All individuals were handled in strict accordance with good animal practice as defined by the current European legislation.

### Sampling

All samples used in this study (S1 Table in Supporting Information) were collected with appropriate permissions from local authorities (see acknowledgments) during several expeditions to the Socotra Archipelago between 30 September–4 November 2007, 22 December 2007–26 February 2008, 20 December 2008–22 February 2009, 15 March–9 April 2010, 14 March–11 April 2013, and 28 February–18 March 2014. Reptile samples were collected during both nocturnal and diurnal transects of 45 min of duration on average, made by two to seven herpetologists in 110 sample stations, covering the entire archipelago in latitude, longitude and altitude (Fig 1). Animals were caught by hand or with nooses and searches were conducted for both active and inactive animals (turning rocks, looking inside barks and fissures). The most resembling species are allopatric of live at different altitude levels, so it is easy to assign them to species, excluding the possibility of misidentification. Even so, photos of every specimen were taken to recheck identification in case of doubt. Special efforts were made to include the whole distributional range of each one of the 31 species. In order to optimize the sequencing effort, sample sites for DNA barcoding were selected using a 10x10 km square grid. For the sympatric species with already recognized cryptic diversity (*Hemidactylus inintellectus*, *Hemidactylus pumilio* [24] and *Pristurus sokotranus* [28] three individuals instead of one were randomly selected at each locality (S1 Fig).

### DNA extraction, amplification and sequencing

Total genomic DNA was extracted from alcohol-preserved tail muscle collected from living specimens, or from vouchers following a standard saline method [31]. For samples in which this protocol did not work, genomic DNA was extracted using the Speedtools Tissue DNA Extraction Kit (Biotools B&M Labs S.A.) following manufacturer’s instructions. All samples were amplified for the COI gene using three different pairs of primers; two of them specifically designed for this project due to amplification problems in *P*. *sokotranus*, *Pristurus obsti* and *Pristurus guichardi*. Primers, PCR conditions and source references are detailed in S2 Table. Purification and sequencing of PCR products were carried out by Macrogen Inc., Amsterdam. Chromatograms were checked manually, assembled and edited using Geneious Pro v.6.1.3 (Biomatters Ltd.). Sequences were aligned using the online version of MAFFT v.7 (http://mafft.cbrc.jp/alignment/server/) with default parameters and translated into amino acids using the vertebrate mitochondrial genetic code and neither stop codons nor gaps were observed. The alignment was submitted to a substitution saturation test in DAMBE v5.3.108 [32].

### Distance-based analyses

Intra-specific and intra-generic genetic distances (*p*-distance) were calculated using MEGA6 [33]. Since there is some controversy for estimating inter-specific genetic divergences [7], these were calculated through pairwise distance comparisons between all individuals. This allowed exploring not only the mean values, but also the minimum and maximum inter-specific distances.

To determine the specimen identification success through DNA barcodes, three different query identification analyses (‘best match’, ‘best close match’ and ‘all species barcodes’) were conducted in SpeciesIdentifier v1.7.8 following the criteria stated by Meier *et al*. (2006) [34] to distinguish between successful, ambiguous or misidentified sequences. In the case of ‘best close match’ and ‘all species barcodes’ identification success or failure was assessed using a series of distance thresholds (1%, 3%, 6%, 9% and 14.38%). The reason for choosing these thresholds are: (i) 1% represents the standard cut-off value fixed by the BOLD (Barcode of Life Data) system [35], (ii) 3–9% thresholds have been used in similar studies of reptiles [36,37], and (iii) 14.38% represents the distance below which 95% of all intra-specific pairwise distances were found in the present dataset. We also tested whether species retained unique barcodes when using the consensus sequence for all available conspecific sequences.

Frequency distribution histograms for all conspecific and all heterospecific pairwise distances at species, genus and higher taxonomic levels (Serpentes, Scincoidea, Lacertoidea and Gekkota [38]; were built to depict barcoding gaps. These divergences were then used to explore cryptic diversity among groups. Potential cryptic diversity was detected when intra-specific genetic distance exceeded the inter-specific genetic distance. Although this approach is very effective for species discovery, some criticism exists about its performance on specimen identification [39], especially because of the use of a global barcoding gap, i.e., a fixed distance threshold for all species. For this reason, for each species with more than one sequence, the maximum intra-specific distance (distance to the furthest conspecific) was plotted against the minimum inter-specific distance (nearest neighbour) in a dotplot to explore local barcoding gaps.

The ability of DNA barcoding to delimit clusters of species was assessed on SpeciesIdentifier v1.7.8 (http:/taxondna.sourceforge.net/) [34] by measuring the level of overlap (total and 90%) between intra- and inter-specific variability with a minimum of 300, 400, 500 and 600 base pairs (bp) overlap for all genetic distance thresholds (see below). This program makes pairwise comparisons for all sequences, and clusters together those sequences having *p*-distances within a fixed threshold value. For all resulting clusters, it was firstly verified whether the largest observed distance exceeded the threshold and the number of threshold violations, and secondly, whether the number of clusters found corresponded to the number of currently accepted taxonomic species. Following Hendrich *et al*.’s taxonomic accuracy [40] was calculated as the number of perfect clusters (i.e., clusters comprising all sequences of one species and only those) relative to the number of currently recognized species of Socotran reptiles.

### Tree-based analyses

As recommended by other authors [35], a tree-based approach, including Neighbour-joining (NJ), Maximum-likelihood (ML) and Bayesian inference (BI), was also used for testing the specimen identification success of the barcode library and to explore the effectiveness of the COI molecule in recovering the phylogenetic relationships of Socotran reptiles, in comparison with other studies performed with more genes. NJ analyses were conducted in MEGA6. Evolutionary distances were computed using the p-distance (number of base differences per site). Best fit partitioning schemes and substitution models for ML and BI analyses were identified using PartitionFinder v1.1.0 [41] with the following searching criteria: branch lengths = linked; models = raxml or models = mrbayes, depending if the output was used for ML or BI analyses; model_selection = BIC; three datablocks (one for each codon position of the COI gene); and search = all. In both ML and BI analyses, the optimal gene partitioning scheme was all three codon positions together (single partition for the COI) and the selected model was the GTR+G+I. Maximum-likelihood trees were generated in RaxML v7.0.3 [42] as implemented in raxmlGUI [43] with a heuristic search using 100 random addition replicates and 1,000 bootstrap iterations. Bayesian inference analyses were performed with BEAST v1.8.0 [44]. Three independent runs of 5x10^7^ generations were carried out, sampling at intervals of 10,000 generations, producing 5,000 trees each. Models and prior specifications were as follows (otherwise by default): model of sequence evolution for the single COI partition GTR+I+G; Relaxed Uncorrelated Lognormal Clock (estimate); Speciation Coalescent Constant Size process tree prior for the phylogenetic reconstruction; random starting tree; base substitution prior Uniform (0, 100); alpha prior Uniform (0, 10). Posterior trace plots and effective sample sizes (ESS) of the runs were monitored in Tracer v1.5 (http://beast.bio.ed.ac.uk/Tracer) to ensure convergence. Results of individual runs were combined in LogCombiner discarding 10% of the samples and the ultrametric tree was produced with TreeAnnotator (both provided with the BEAST package).

Specimen identification success was initially assessed as described by Hebert *et al*. (2003a) [1] and then using the ‘revised tree-based identification criteria’ developed by Meier *et al*. (2006) [34]. Both methods focus on whether individuals from the same species cluster together or not. According to the first authors, specimen identification was considered successful when all conspecific sequences clustered together in a unique cluster. Misidentification occurred when sequences from a single species were found in more than one cluster, whereas ambiguities occurred when species had a single sequence. According to latter authors, however, a query sequence was considered correctly identified if it was included within a conspecific polytomy or cluster (regardless whether all its conspecific sequences were included or not). In this case, identification was ambiguous when a species had only one or two sequences, or when those sequences formed a sister group to a cluster of conspecific sequences. Finally, sequences within allospecific clusters were considered misidentified.

The number of species-like units (species discovery) present in the DNA barcoding library was also tested using the generalized mixed Yule coalescent (GMYC) approach [45]. This method, in contrast to the distance-based analyses described above, relies on phylogenetic information for cluster delimitation. It identifies species boundaries as a shift in branching rates on a phylogenetic tree that contains multiple species and populations. Here, a ML approach was used for estimating a certain threshold for the shift from inter-specific to intra-specific branches on a phylogenetic tree [45,46]. Since in deep phylogenies the evolutionary rate between groups is not always homogeneous, a single threshold may not reflect the variety of genetic divergence among taxa; therefore several tests and three-based approaches were performed allowing first single and then multiple thresholds over time across trees with three different datasets of the 380 sequences manually generated: A—Squamata—included all 380 barcoded sequences in the same analysis (model of sequence evolution GTR+G+I); B—higher taxa—included independent GMYC analyses for the following higher taxa: Serpentes (suborder, N = 37, GTR+I+G), Scincoidea (suborder, N = 24, TPM1uf+G), Lacertoidea (suborder, N = 32, GTR+G), Gekkota (infraorder, N = 287, GTR+I+G), an independent GMYC analysis for the higher taxa Iguania (infraorder) could not be carried out as a single genus and species (*Chamaeleo monachus*) occurs in Socotra; C—families—included independent GMYC analyses for the following families of Socotran reptiles: Leptotyphlopidae (N = 14, GTR+G), Scincidae (N = 24, TPM1uf+G), Lacertidae (N = 31, HKY+G), Sphaerodactylidae (N = 139, TrN+I+G), Phyllodactylidae (N = 24, TrN+I), Gekkonidae (N = 124, GTR+I+G), the remaining families (S1 Table) could not be included in the GMYC analyses as all were represented by a single genus and species in Socotra.

Identical sequences were removed before the analyses and the best fit partitioning scheme and substitution model for the BI analysis (single partition for COI for all analyses and respective models listed above) were identified using PartitionFinder v1.1.0 [41] with the same searching criteria as above. The ultrametric trees required for GMYC were generated with BEAST using the same priors and parameters as above. GMYC analyses were conducted using the R-package SPLITS (http://r-forge.r-project.org/projects/splits). Taxonomic accuracy was recorded for each dataset, and also the total number of GMYC clusters for each of the 31 currently recognized species.

## Results

### DNA reference library for the reptiles of the Socotra Archipelago

The length of all aligned sequences (N = 380), except one, were greater than the minimum sequence length required by CBOL's standards (500 bp; www.barcoding.si.edu). The resulting alignment consisted of 294 conserved, 369 variable and six singleton sites. The nucleotide base compositions were: A = 23.8%, C = 29.2%, G = 18.5%, T = 28.5% (GC content = 47.7%).

Results of the substitution saturation test showed that the saturation index was significantly lower than the critical value when performing the analysis for symmetrical topology (*p*-value<0.0001) on fully resolved sites, suggesting little or no saturation.

### Effectiveness of DNA barcoding for specimen identification

**Fig 2:**
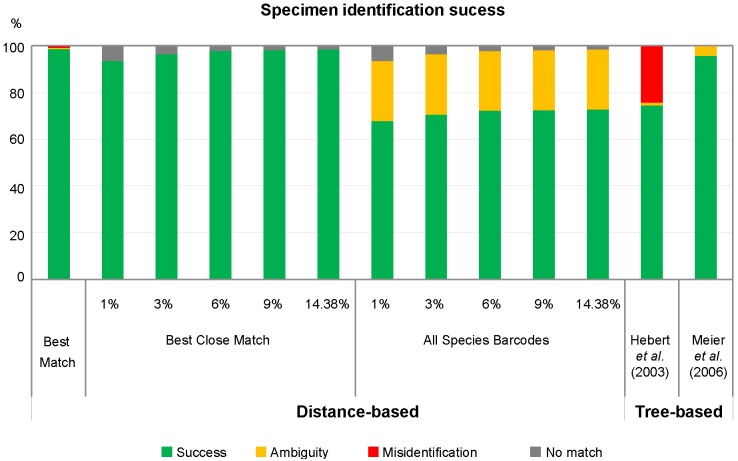
Specimen identification success. Values for both distance-based (‘best match’; BM, ‘best close match’; BCM, and ‘all species barcodes’; ASB—using different distance thresholds) and tree-based (Hebert et al. 2003a; Meier et al. 2006) approaches [1,34].

Distance-based (‘best match’, ‘best close match’, ‘all species barcodes’) and tree-based analyses (criteria of Hebert *et al*. 2003a and Meier *et al*. 2006) [1,34] presented different rates of success of identification, ambiguity, misidentification, and no match (Fig 2).

Regarding the distance-based criteria, identification success was moderate to high (68–99%). The ‘best match’ criterion reached the best barcode match at 99%. Only four sequences, for species represented by a single sequence each, were identified as ambiguous or misidentified as their assignation with a conspecific sequence was impossible. Nevertheless, the two congeneric species were assigned to the same genus. The ‘best close match’ criterion yielded an identification success rate from 94–99% depending on the threshold, with no ambiguous or misidentified queries for any of the thresholds (1–6% ‘no match’ for the same 4 sequences). Finally, for the ‘all species barcodes’ criterion, a moderate identification success rate was obtained, ranging from 68–73% when using the 1% and the 14.38% distance threshold, respectively. Unidentified queries remained the same as in the ‘best close match’ and there were no misidentified queries (Fig 2).

According to the tree-based criteria of Hebert *et al*. (2003a) [1], 75% of queries representing 83.87% of all 31 species were successfully identified (Fig 2). Some of the failed sequences were ambiguous (1%) and remained unidentified due to the lack of conspecifics, but most of them were misidentified (24%) since they failed to form species-specific clusters. According to the criteria of Meier *et al*. (2006) [34], however, the proportion of successfully identified query sequences was higher (96%) and the proportion of ambiguous sequences was lower (4%) than the criteria of Hebert *et al* (2003a). Furthermore, not a single sequence was misidentified because this criterion does not require monophyly (i.e., sequences from *P*. *sokotranus* can be identified even though they are in different clades). Additionally, we found that each species had unique consensus barcodes.

### Exploration of cryptic diversity using DNA barcoding

The level of intra-specific genetic variability (*p*-distances) varied greatly depending on the taxa (0–17.10%; average = 7.04%; Table 1). As expected, variability was higher at the intra-generic (inter-specific) level (3.13–24.15%; average = 18.50%). Unexpectedly high levels of intra-specific divergence (>9%) were detected in seven of the 27 species (one skink, *H*. *simonyi*, and six geckos, *P*. *insignis*, *P*. *sokotranus*, *H*. *trachyrhinus*, *H*. *riebeckii*, *H*. *pumilio* and *H*. *inintellectus*), suggesting the presence of cryptic diversity in those groups (Table 1).

**Table 1:** Genetic divergences.

Species	Intra-specific pairwise distance (%)			Intra-generic pairwise distance (%)		
	Min.	Mean	Max.	Min.	Mean	Max.
*Chamaeleo monachus* ¥	0.00	0.32	0.75	-	-	-
Xerotyphlops socotranus*¥	-	-	-	-	-	-
*Myriopholis filiformis*	0.00	0.00	0.00	14.33	.37	16.44
*Myriopholis wilsoni*	0.00	4.20	6.94		15	
*Myriopholis macrura*	0.00	1.23	2.26			
*Hemerophis socotrae* ¥	0.00	0.30	0.45	-	-	-
*Ditypophis vivax* ¥	0.00	1.54	3.17	-	-	-
*Hakaria simonyi* ¥	0.90	4.30	9.05	-	-	-
Trachylepis cristinae*	-	-	-	17.95	18.14	18.40
*Trachylepis socotrana*	0.00	0.74	1.96			
Pachycalamus brevis*¥	-	-	-	-	-	-
*Mesalina kuri*	0.00	0.72	1.36	13.73	14.14	14.93
*Mesalina balfouri*	0.00	0.57	1.81			
*Pristurus abdelkuri*	0.00	0.43	0.77	3.13	18.47	24.15
*Pristurus insignoides*	0.00	0.69	1.36			
*Pristurus insignis*	0.00	7.08	17.10			
*Pristurus guichardi*	0.00	0.93	1.41			
*Pristurus obsti*	0.00	0.38	0.78			
*Pristurus sokotranus*	0.00	8.59	15.48			
*Pristurus samhaensis*	0.00	0.14	0.33			
*Haemodracon trachyrhinus*	0.30	5.53	12.67	18.77	21.35	22.47
*Haemodracon riebeckii*	0.00	6.25	11.92			
*Hemidactylus pumilio*	0.00	5.39	14.00	7.84	18.63	23.03
Hemidactylus flaviviridis*	-	-	-			
*Hemidactylus robustus*	0.33	1.41	2.49			
*Hemidactylus forbesi*	0.15	0.15	0.15			
*Hemidactylus oxyrhinus*	2.26	2.26	2.26			
*Hemidactylus homoeolepis*	0.15	2.54	4.83			
*Hemidactylus granti*	0.00	0.12	0.30			
*Hemidactylus dracaenacolus*	0.00	1.36	2.41			
*Hemidactylus inintellectus*	0.00	7.02	13.27			
**Total genetic divergence**	**0.00**	**7.04**	**17.10**	**3.13**	**18.50**	**24.15**

Minimum (Min.), mean, and maximum (Max.) genetic divergences (*p*-distances) within species and genera of Socotra reptiles.

*Species represented by single sequences.

^¥^ genera represented by single species.

**Fig 3:**
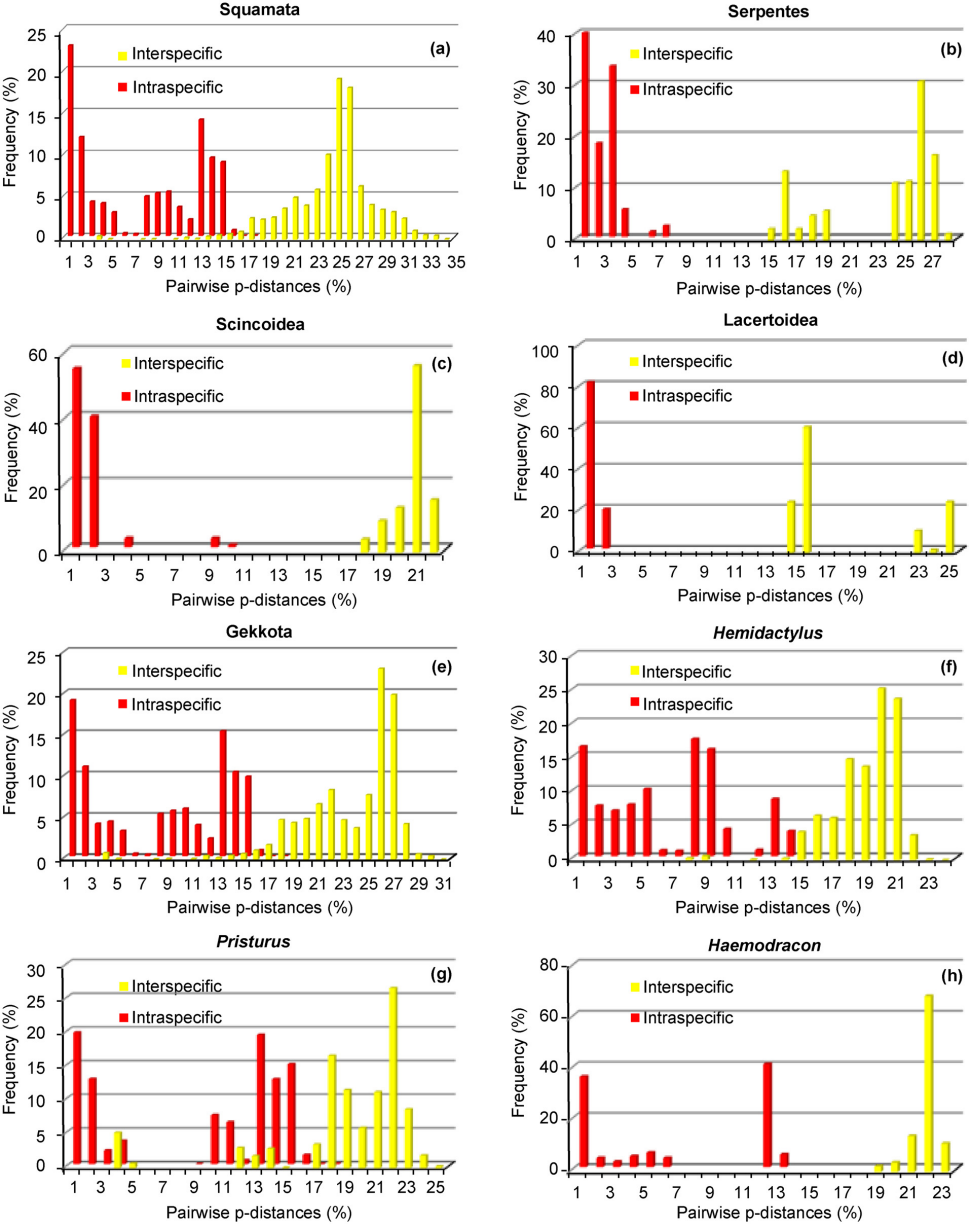
Frequency distribution histograms of all pairwise *p*-distances of the Socotran reptiles for COI. Distances are given at different taxonomic levels: (a) all Squamata species; (b–e) higher taxonomic groups: Serpentes, Scincoidea, Lacertoidea and Gekkota; (f–h) all gecko genera *Hemidactylus*, *Pristurus* and *Haemodracon*.

**Fig 4:**
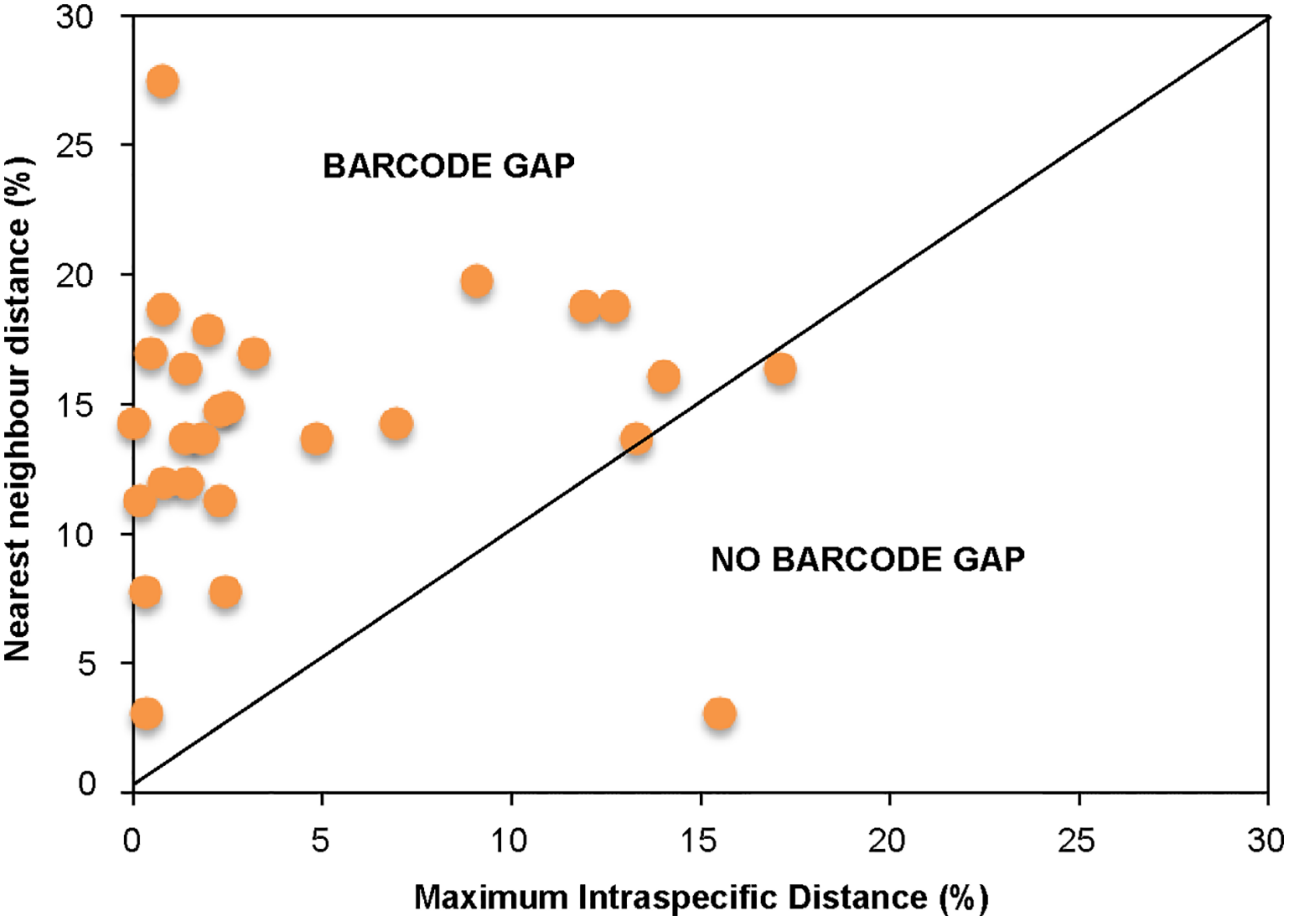
Comparison of maximum intra-specific divergence with nearest-neighbour distance for each species in the dataset. Points above the 1:1 line indicate the presence of a local barcoding gap. Note that only 27 points are displayed as four species have single sequences.

Only some frequency distribution histograms of all pairwise *p*-distances at different taxonomic levels presented barcoding gaps (Fig 3). The histogram for all 380 sequences shows a great overlap (13.97%) between intra and inter-specific *p*-distances (3.13–17.10%; Fig 3a) and confirms that, considering the current taxonomy, a global barcoding gap does not exist for the reptiles of the Socotra Archipelago. A closer examination of the frequency distributions grouping by higher taxonomic level (Fig 3b–3e), suggests that Gekkota is the problematic group, as all the others present a barcoding gap (between 6.94–14.33% in Serpentes, 9.05–17.95% in Scincoidea and 1.80–13.73% in Lacertoidea). Further examination of the Gekkota histograms show that *Hemidactylus* (Fig 3f) and *Pristurus* (Fig 3g) are the problematic genera, presenting great overlap of their frequency distributions (between 7.84–14.00% and 3.13–17.10% respectively), contrary to the genus *Haemodracon* that shows a clear barcoding gap (between 12.67–18.77%; Fig 3h). Although some of these intra-specific divergence values are high, for all species except two (*Pristurus insignis* and *P*. *sokotranus*), they were still lower than the nearest-neighbour distances (Fig 4). This explains the presence of a local barcoding gap for 25 out of the 27 species for which it could be calculated.

In the cluster analyses, the same values were found for total and average overlap between intra-specific and inter-specific variability of COI sequences, independently of the overlap length. This resulted in a high percentage of pairwise distances falling into this overlapping interval (33.61–35.59% depending on the overlap length). Thus, analyses were only conducted with a 300 bp overlap of COI sequences. The largest observed intraspecific distances exceeded the fixed value for all thresholds (1%→2.07%; 3%→3.92%; 6%→6.93%; 9%→11.05%; 14.38%→17.94%) and a certain number of clusters had threshold violations (2.43–20.83% of the clusters; Table 2). This resulted in the identification of 24–78 species-like clusters, depending on the distance threshold used (the larger the threshold, the lower the number of clusters). The number of clusters never coincided with the number of currently accepted species. The best taxonomic accuracy was achieved with the 6% threshold, with a total of 41 clusters, 19 of which (61.29%) coincided with the currently accepted taxonomy (Table 2).

**Table 2:** Taxonomic accuracy.

Delimitation method	Threshold/ Dataset	Clusters	Taxonomic accuracy			Threshold violations	
			N	%	Max.	N	%
Distance-based (Pairwise distance threshold)	1%	78	11	35	1	11	14
	3%	52	18	58	1	6	12
	6%	41	19	61	2	1	2
	9%	36	18	58	2	3	8
	14.38%	24	12	39	3	5	21
Tree-based (GMYC)	A—Squamata	67	17	55	-	-	-
	B—Higher taxa	68	18	58	-	-	-
	C—Families	58	18	58	-	-	-

Obtained for both distance-based and tree-based delimitation methods. The total number of clusters obtained by each threshold or dataset are detailed. Taxonomic accuracy/ threshold violations are calculated as the number (N) of perfect clusters (i.e., species corresponding/ non-corresponding to only one cluster and vice-versa), the percentage (%) relative to the number of total species of the Socotra Archipelago or the maximum number of species-like units per cluster (max.). See Material and Methods for further details.

**Fig 5:**
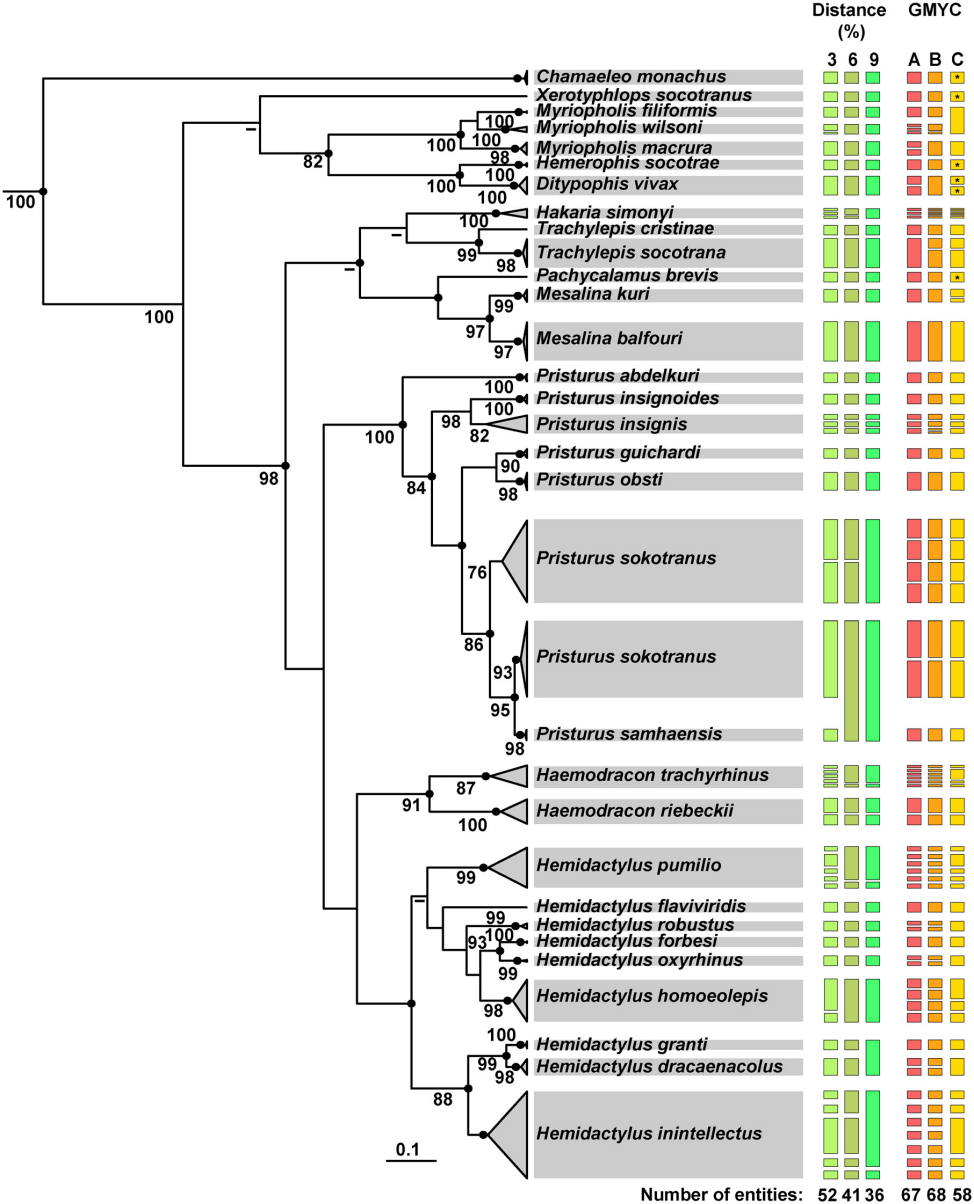
Bayesian COI tree for all the reptiles of the Socotra Archipelago. Species delimitations using three distance thresholds with higher taxonomic accuracy (3%, 6% and 9%) and GMYC using three different datasets: A—including all 380 barcoded sequences in the same analysis; B—including independent analyses for Serpentes, Scincoidea, Lacertoidea and Gekkota; C—including independent analyses for the following families: Leptotyphlopidae, Scincidae, Lacertidae, Sphaerodactylidae, Phyllodactylidae, Gekkonidae; *indicates clusters that were depicted as in A because taxa are monoespecific or monogeneric. See Material and Methods for further details. Black dots indicate posterior probability values ≥0.95. Bootstrap values ≥70% of the ML analysis are shown next to the nodes.

A phylogenetic representation of the Socotran reptiles based on the distance thresholds with higher taxonomic accuracy (3%, 6%, and 9%) depicts different delimited species clusters (Fig 5). Based on the 3% threshold, many species were divided into more than one cluster, most of them belonging to Gekkota. When the threshold was increased to 6%, the number of clusters was much lower, as *Pristurus samhaensis* clustered together with some specimens of *P*. *sokotranus*. Finally, cluster numbers diminished even further with the 9% threshold, however two morphologically and ecologically well-defined species, *Hemidactylus granti* and *Hemidactylus dracaenacolus*, clustered together (Fig 5).

Using the tree-based criteria more clusters were identified (Table 2; Fig 5). For each dataset, the generalized mixed Yule coalescent (GMYC) analyses resulted in a varying number of entities depending on the type of threshold used. Taxonomic accuracy was generally much lower when using multiple-thresholds than a single-threshold, regardless of the dataset used, as already confirmed in similar studies and simulated data [47]. Therefore, only results using single-thresholds are presented. GMYC defined 67 entities using approach A, with 54.84% of all 31 entities corresponding to accepted species (Table 2). The number of clusters recovered with approach B and C was 68 and 58, respectively. Despite this, both B and C approaches had the same taxonomic accuracy (58.06%; Table 2). There were not many differences between the putative species supported by approaches A and B. Both supported a relatively higher number of putative species than accepted in 12 of the species (Fig 5). Results from approach A and B differed in that they consider *Myriopholis macrura* and *Ditypophis vivax* to be formed by two putative species and *Trachylepis socotrana* by only one (Fig 5). Following the approach C, the number of putative species in all cases was lower or equal compared to other approaches, with the single exception of *Mesalina kuri* that was split in two putative species. *Myriophilis filiformis* and *M*. *wilsoni* were, however, joined (Fig 5).

### Robustness of phylogenetic inference and species delimitation using COI

All phylogenetic trees (Neighbour-joining, NJ, Maximum-likelihood, ML, and Bayesian inference, BI) displayed similar topologies and differed only in the reconstruction and support values of some nodes (Fig 5, S2 and S3 Figs). Outgroups were not included in the analyses and in the case of the BI the Bayesian method was used for inferring the root of the phylogenetic tree. The chamaeleonidae *Chamaeleo monachus* was recovered as sister to all the other squamates included in the BI analysis and therefore the sister taxa relationship between snakes and chamaeleonids was not supported. For comparison purposes with the BI tree, in the ML and NJ trees *Chamaeleo monachus* was used to root the tree. In contrast with ML, NJ and BI recovered both Serpentes and Scincoidea (with high support in the case of NJ) as monophyletic. Only BI recovered amphisbaenians and lacertid lizards as sister taxa with high support, and generally nodes were better supported than with NJ or ML, with the exception of the node supporting *Haemodracon* and *Hemidactylus* as sister taxa (Fig 5, S2 and S3 Figs). The ML tree performed poorly at the deepest nodes (S2 Fig). Moreover, the phylogenetic relationships within the genus *Hemidactylus* were different from those obtained in the NJ and BI. While in the ML tree *H*. *pumilio* formed a clade with *H*. *inintellectus*, *H*. *dracaenacolus* and *H*. *granti*, in the NJ and BI trees, it emerged as sister group to a clade formed by *H*. *flaviviridis*, *H*. *robustus*, *H*. *forbesii*, *H*. *oxyrhinus*, and *H*. *homoeolepis*.

## Discussion

Although some COI gene amplification problems in reptiles were reported in the past, we achieved successful amplification and sequencing as a result of freshly collected material and the design of new primers for certain problematic taxa. The quality of our DNA reference library is based on extensive sampling (Fig 1). Its strategic design was to accomplish the first large-scale study covering all recognized species within a taxonomic class and their entire distributions, so that the intra-specific diversity would be covered [36]. We expect such approach to be useful for future taxonomic and conservation work. Ecological research using unidentified samples of these species (e.g., faecal pellets) will also be possible.

The high levels of intra-specific divergence found within some species (e.g., *P*. *sokotranus*) show that the COI gene can differ considerably between individuals of the same species (Table 1; S1 Fig). Despite this, we recovered a single DNA consensus barcode sequence for each species (S1 Appendix). Conversely, the low inter-specific variation observed between some specimens of *P*. *sokotranus* and *P*. *samhaensis* shows that some taxonomic rearrangements may be needed. The high overlap between intra- and inter-specific genetic distances of Socotra reptiles was expected, and coincides with numerous studies, even those performed in well-sampled groups with closely related-species and stable taxonomies [4,6,8,39,48]. The taxonomy of Socotran reptiles is far from being complete, as in recent years new species have been described [26], and cases of paraphyly have been identified, which are generally avoided in recent taxonomic reviews. Additionally, it is well known that genetic divergences vary among species due to population size, mutation rates, or biogeographic history, etc. [49]. The absence of a global barcoding gap, which is required for species discovery, does not imply that COI cannot be used for specimen identification purpose, that instead generally relies upon the presence of a local barcoding gap [16,36]. The latter was detected for most of the analysed species (Fig 4), suggesting that DNA barcoding is an effective tool for specimen identification.

Using all criteria the specimen identification success rate was moderate to high in this study. The ‘best match’ and the ‘best close match’ approaches reported very high success rates even when applying a threshold of 1%. However, the ‘all species barcodes’ had lower identification success than other distance-based and tree-based criteria and a high proportion of ambiguous sequences. This is because ‘all species barcodes’ is centred on the premise that all sequences from conspecifics are more similar to each other than any of them are to sequences of heterospecifics, which is not the case of *P*. *sokotranus*. This is in accordance with the results obtained with Hebert *et al*. (2003a)’s tree-based criteria [1] that classified these sequences as misidentifications, as *P*. *sokotranus* is paraphyletic in all inferred phylogenetic trees. In the present dataset there are also some species with less than two conspecific sequence matches, which hampered successful identifications with the latter distance approach. These results suggest that, in general, ‘all species barcodes’ and Hebert *et al*. (2003a)’ s approaches [1] are better at detecting taxonomic problems, while ‘best match’ and ‘best close match’ methods reported high success rates despite them. We emphasize that cases of paraphyly do not prevent the identification of specimens unless they share haplotypes, which is not this case. Such cases also highlight the importance of comprehensive sampling (across different populations and geographic regions) without which some species pairs in our dataset may have appeared as reciprocally monophyletic, leading to DNA barcoding performance misinterpretations.

Through comprehensive sampling, this study reveals that many of the pairwise distances in Socotran reptiles fall into the region overlap between intra- and inter-specific genetic variability (35.59%; Fig 3a). Results of the distance and tree-based methods suggest the existence of undescribed diversity, and that reptile species richness on the archipelago may be under-estimated by 13.8–54.4%. These results are supported by the fact that most deeply divergent lineages present geographic structure (Fig 5 and S1 Fig). Mean intra-generic divergence was much higher (18.50%) than the mean value found for COI of vertebrates (9.6%) [1], which is also an indication that the number of species of Socotran Reptiles is underestimated. Depending on the delimitation method used, the total number of species-like clusters detected varied. Yet, this number never corresponded to current taxonomy (Table 2; Fig 5), stating the need for detailed taxonomic studies. Using any distance-based approach, high error rates were evident based on the current taxonomy or unrealistic merging of well-differentiated species for higher thresholds (Table 2; Fig 5). The choice of a threshold is somewhat arbitrary as we were unable to identify an ideal distance for delimitation of all currently accepted species. Another serious problem is that fixed thresholds are logically impossible to maintain because pairwise distances between a set of samples do not have to be similar in order to all be included in the same cluster [34]. The GMYC approach uses inferred evolutionary trees instead of distances for optimizing thresholds, so it avoids the problems that underpinned DNA barcoding and therefore leads to a more robust, integrated, and reliable method of species richness estimation [36,46]. Even though different estimations are returned when different datasets are used (Table 2; Fig 5), it gives a boundary of how many species-like units are unveiled, which can then be estimated using congruence approaches.

In general, phylogenetic relationships inferred using COI are consistent with previous studies using several mitochondrial and nuclear molecular markers but differ in some of the deepest nodes, with the currently accepted phylogenies of Squamata [38,50]. The robustness of this barcode tree is supported by its congruence with the phylogeny of *Pristurus* inferred with five markers (including four nuclear) [28]. Paraphyly within *P*. *sokotranus* was observed in all trees (Fig 5, S2 and S3 Figs). In the ML tree (S2 Fig), relationships observed within species of the genus *Hemidactylus* match well with the results obtained using four markers (including one nuclear) [24], whereas in the NJ and Bayesian tree (Fig 5), phylogenies are also compatible with the ones previously obtained for *Trachylepis* skinks [26], *Haemodracon* geckos and *Myriopholis* snakes (unpublished data). These results suggest that COI has great phylogenetic signal as stated by Hebert *et al*. (2003a) [1].

In addition, this marker seems to recover GMYC cluster numbers similar to a previous study on the *Hemidacylus* that used three different mitochondrial markers when the analyses were run at the family level (column C in Fig 5) [24], although it was demonstrated that GMYC significance decreases when reducing the number of species and tree depth within partitions [51]. The latter study also recovered five putative species for *H*. *pumilio*, one for *H*. *forbesi*, three for *H*. *homoeolepis*, one for *H*. *dracaenacolus* and five for *H*. *inintellectus*. Our results only differed in that we recovered only one cluster for *H*. *granti* and *H*. *oxyrhinus*. In general, our results show that despite the reduced length of COI sequences, this molecule is very useful for detecting intra-specific diversity and flagging species discovery, confirming other results from simulated data [47].

Although short-length markers are often time and cost-effective proxies for inferring phylogenetic trees [16], they are frequently not representative of the full evolutionary history of species [48]. Occasionally, mitochondrial DNA evolutionary relationships may disagree with nuclear DNA inferences due to incomplete lineage sorting or introgression [52]. Thus, phylogenetic inference should be validated by independent lines of evidence, especially in young species radiations [52]. Therefore we propose that the taxonomy of Socotran reptiles should be revised in light of an integrative framework incorporating multiple loci, population genetics, morphological, and/or ecological data, similar to other work carried out in Arabia [25,28,53,54]. Only after the recognition of the appropriate units and scales for conservation planning could a proper management plan be sketched for Socotran reptiles.

Considering that currently 35% of Socotran reptiles are classified as threatened, Near Threatened, or Data Deficient [55], the results of this work should be confirmed, as they have major implications for species conservation, such as a change of distribution range and consequently of conservation status. A taxonomic revision is needed because, despite previous evidence of high levels of genetic diversity within many reptile species, conservationists and politicians still focus their effort around named species, as do data compendia such as the IUCN Red List. Given the importance for robust conservation actions in Socotra, we hope that public access to DNA barcodes will ultimately boost taxonomic work. It should be stressed that Socotran reptiles are the target of incipient illegal pet-trade, more specifically the gecko *Haemodracon riebeckii* and the Near Threatened and CITES species, the chameleon *Chamaeleo monachus* [17,55]. Quick identification of specimens and biological material by airport/ ports authorities may preclude the growth of the illegal pet-trade and the introduction of non-endemic species, while in general, it would assist in managing the whole reptile community for long-term sustainability. Hence, this library will be useful for enforcing laws related to biological resources and triggering monitoring advice from the IUCN. Apart from this, it will also allow non-experts to detect and monitor the translocation of endemic species on the archipelago (e.g., *Pristurus abdelkuri* in Socotra Island) and exotic species (e.g., *Hemidactylus robustus* and *H*. *flaviviridis*) that were newly introduced to the islands [27]. Considering the cost-efficiency advantages of complete barcoding demonstrated in this model study, we recommend applying it to other biodiversity hotspot areas given the urgency to promote conservation actions in these locations.

## Supporting Information

S1 Appendix Consensus Barcode sequences of the 31 reptiles species of Socotra. (DOCX)

S1 Fig Species maps showing the localities and phylogenetic relationships of all Socotran reptiles. A total of 380 specimens of included in this study. White dots represent bibliographic and new distribution records, and green stars sampled specimens. Black dots on trees indicate posterior probability values ≥ 0.95, and values next to the nodes Maximum Likelihood bootstraps ≥ 70%. Maps were drawn using DIVA-GIS v.7.5 (available at http://www.diva-gis.org). Photos reprinted [27] with permission from Edoardo Razzetti and Roberto Sindaco.
(DOCX)

S2 Fig Maximum likelihood phylogenetic tree for all reptiles of the Socotra Archipelago. Phylogenetic relationships based on COI gene. Bootstrap values >70% are shown next to the nodes.
(DOCX)

S3 Fig Neighbour-Joining tree for all reptiles of the Socotra Archipelago. Bootstrap values ≥70% are shown next to the nodes. See Material and Methods for further details.
(DOCX)

S1 Table Details of the samples. Taxonomic information and number of COI sequences amplified (N) of endemic and introduced reptile (*) species present (•) in the Socotra Archipelago. Question mark (?) stands for unchecked bibliographic records following recent work [27].
(DOCX)

S2 Table Details and amplification conditions of COI primers used in this study. (DOCX)
