# Supplementary material for: Unexpectedly High Levels of Cryptic Diversity Uncovered by a Complete DNA Barcoding of Reptiles of the Socotra Archipelago
Source: PLoS One. 2016 Mar 1;11(3):e0149985. doi: 10.1371/journal.pone.0149985 (PMC4772999; doi:10.1371/journal.pone.0149985)
Supplement: S1 Appendix — (DOCX) [file pone.0149985.s001.docx]

**Supporting Information**

**Unexpectedly high levels of cryptic diversity uncovered by a complete DNA barcoding of reptiles of the Socotra Archipelago**

**Raquel Vasconcelos, Santiago Montero-Mendieta,**

**Marc Simó-Riudalbas,**

**Roberto Sindaco,**

**Xavier Santos,**

**Mauro Fasola,**

**Gustavo Llorente**

**Edoardo Razzetti**

**Salvador Carranza**

**S1 Appendix.** **Consensus Barcode sequences of the 31 reptiles species of Socotra.**

*Chamaeleo monachus*

ATCGGCACAATATACTTTCTATTTGGACTCGCTGCAGGACTTGTTGGGGCCACTTCAAGCCTGCTAATACGCACAAAACTCAGTCAACCTGGGTTCTCTCTGGGRGACGATCATGCTTATAATGTTTTAATYACCCTTCACGGATTAACCATAATCTTCTTCATAGTCATACCAATCATGATTGGAGGGTTCGGAAATTGACTTGTACCCTTAATACTTGGAGCACCYGATATGGCTTTCCCACGTATAAACAACATGAGTTTTTGACTTMTTCCACCATCATTYATACTTCTACTAGCATCATCAAAAACAGGAACCGGAGTTGGAACAGGATGAACTATTTATCCRCCACTATCKGGAAATATAGCACACTCAGGACCCTCAATAGACCTAGCAATTTTTTCACTTCACCTAGCTGGAATTTCATCTATTCTTGCTTCAATTAACTTTATTACAACAAGCATTAACATAAAACCACACCACATAGTACTATACAACCTGCCTCTATTCGTTTGATCAGTTATACTAACTGCAATCCTACTAATCCTAGCCTTACCTGTATTAGCTGCAGCCATTACAATACTCTTAACAGATCGAAATCTAAACACAGCATTCTTTGATCCCGTAGGCGGCGGAGATCCGGTCCTATTTCAACACCTATTC

*Ditypophis vivax*

ATTGGAACCCTATACCTRTTATTCGGGGCCTGRTCCGGCTTGATTGGGGCATGCCTRAGCATACTRATACGAATAGAGCTAACCCAACCAGGNTCCCTCCTGGGCAGCGATCAGATTTTTAACGTMTTAGTGACAGCCCACGCATTCGTGATAATTTTCTTCATGGTCATRCCAATCATGATYGGCGGATTCGGTAACTGATTAATCCCCCTTATAATCGGAGCCCCAGACATGGCTTTCCCACGAATAAACAATATAAGTTTTTGACTRCTACCACCRGCCCTCCTCCTACTCCTATCCTCCTCATAYGTAGAAGCCGGTGCCGGCACAGGCTGAACCGTATAYCCCCCCYTATCTGGAAATCTTGTACACTCKGGCCCATCAGTTGATCTAGCAATTTTCTCYCTTCATYTGGCTGGGGCCTCCTCCATCCTGGGAGCAATTAACTTCATTACYACYTGCATCAATATAAAACCTAAACCYATGCCAATATTTAACATCCCMTTATTTGTRTGRTCAGTCCTAATTACAGCCATCATACTACTCCTCGCCCTGCCAGTCCTTGCCGCTGCAATCACCATACTTCTAACCGACCGAAACCTAAACACATCYTTCTTTGAYCCYTGCGGAGGGGGGGACCCGGTCCTATTCCAACACCTATTC

*Haemodracon riebeckii*

ATCGGAACTCTTTAYTTCATTTTMGGGGCCTGRGCYGGMATGGTRGGAATGGCRCTWAGCCTTTTAATCCGCGCCGAACTMRGCCAGCCCGGCTCTYTRCTWGGCGAYGAYCARRYCTACAAYGTGRTCGTMACCGCCCAYGCCTTTGTAATAATTTTCTTYATAGTRATRCCCGTAATAATTGGGGGGTTTGGTAACTGACTGGTGCCCCTWATAGTMGGGGCACCWGAYATGGCCTTCCCRCGRATAAACAACATRAGCTTCTGRYTGCTTCCCCCCGCACTCCTRCTAYTMCTAGCTTCTTCCGGYATTGARGCCGGTGCCGGCACCGGCTGAACCGTGTACCCACCCCTRGCATCAAACYTRGCCCATGCCGGRGCATCTGTTGACCTRGTCATCTTCTCCCTRCACCTRGCYGGCRTCTCSTCYATYCTRGGGGCYATTAAYTTCATCACCACCTGCATCAATATRAAATCYWCCTCTCTRTCMCAATAYAACACCYCYYTGTTTGTCTGATCYGTDTTAATTACRGCCATCCTRCTCCTYCTAGCACTCCCAGTRCTYGCAGCAGGRATCACYATRCTCYTGACTGATCGAAACTTAAACACCACCTTTTTTGACCCYGCAGGCGGYGGGGACCCRRTYYTATAYCAACACCTYTTC

*Haemodracon trachyrhinus*

ATYGGCACYYTRTACYTACTCTTTGGYGCSTGRGCWGGYATAGTTGGRATAGCCCTYAGCCTCTTRATTCGWGCMGARYTDAGCCARCCVGGCTCRCTHCTRGGMGAYGAYCARRTCTAYAATGTRGTAGTAACAGCHCATGCRTTCGTAATAATCTTCTTCATAGTVATRCCYGTAATAATYGGWGGTTTCGGCAACTGRYTGGTRCCCMTAATAATTGGRGCMCCAGAYATRGCATTCCCHCGAATRAAYAAYATAAGCTTCTGACTWCTTCCTCCKGCACTTCTYYTACTHCTAGCCTCCTCYGGAATTGARGCYGGTGCCGGYACMGGCTGAACTGTGTAYCCVCCCCTAGCAGCYAAYCTAGCYCATGCTGGYGCATCYGTSGAYTTRGTYATYTTTTCTCTMCAYCTTGCAGGKGTRTCTTCDATCCTYGGGGCYATTAAYTTTATYACSACYTGCRTTAAYATAAAACCYCCKACYYTRTCACAATAYAACACYCCMYTRTTTGTRTGRTCYGTYATRATTACAGCYATCCTYCTCCTACTRGCACTACCAGTSCTAGCRGCKGGYATYACYATRCTTCTCACAGAYCGAAATCTYAAYACYACCTTTTTYGAYCCTGCVGGRGGRGGWGAYCCTGTCTTATACCAACAYCTGTTY

*Hakaria simonyi*

ATYGGCACCYTATATTTAATCTTYGGKGCCTGAGCAGGRATRGTSGGCACAGCAYTKAGCCTTTTAATCCGAGCAGAAYTAAGCCAACCAGGWACACTAYTAGGYGATGAYCAGATTTATAATGTTATTGTAACTGCCCATGCCYTTGTAATAATYTTCTTTATAGTTATGCCTGTAATGATYGGCGGATTTGGTAATTGAYTRGTDCCWCTAATAATYGGVGCCCCAGACATAGCATTTCCMCGAATAAAYAAYATAAGCTTYTGACTMCTCCCYCCCTCYCTAYTRCTRCTACTCGCYTCATCCGGGGTTGARGCCGGRGCCGGYACRGGKTGAACAGTTTATCCRCCKYTGGCAGGTAAYYTRGCACACGCMGGAGCCTCAGTTGACCTWACAATYTTCTCATTACACCTAGCTGGYGTTTCATCAATCYTRGGGGCCATYAACTTCATCACAACCTGTATYAATATAAAACCMCCAACYATATCACAATACCAAACCCCMYTRTTCGTRTGRTCAGTATTAATTACCGCYGTMCTRCTTCTTCTATCYCTTCCAGTGCTTGCKGCKGGAATYACAATACTACTWACAGAYCGAAACCTAAACACCTCCTTCTTTGACCCKGCTGGWGGRGGAGACCCRGTATTATACCARCACCTMTTY

*Hemerophis socotrae*

ATCGGAACCCTATACCTCCTATTTGGTGCATGGTCAGGTCTAATCGGTGCCTGCTTAAGCATTTTAATACGCATAGAACTAACACAGCCGGGGTCACTACTCGGTAGTGATCAAATCTTTAATGTATTAGTTACAGCCCACGCATTTATTATAATTTTTTTCATAGTCATACCTATTATAATCGGTGGGTTTGGAAACTGATTAATCCCAYTAATAATCGGAGCCCCRGATATAGCTTTCCCACGAATAAATAACATAAGCTTCTGACTACTACCACCAGCACTTCTTCTACTCCTATCGTCCTCCTATGTAGAAGCCGGTGCTGGCACAGGATGAACMGTCTATCCACCACTATCAGGGAATCTAGTACACTCCGGCCCGTCGGTTGATCTAGCAATCTTCTCCCTACATCTAGCAGGTGCCTCATCAATTCTAGGAGCAATTAACTTTATTACAACATGCATTAATATAAAACCTAAATCCATACCAATATTTAATATCCCCCTATTTGTCTGATCTGTCTTAATCACTGCTATTATACTACTTCTAGCCCTACCAGTACTAGCAGCAGCAATTACCATACTACTAACTGATCGAAATCTAAACACCTCCTTCTTTGACCCTTGCGGGGGAGGAGATCCTGTACTATTTCAACACTTATTC

*Hemidactylus dracaenacolus*

ATTGGCACCCTATATCTACTGTTTGGCGCCTGRGCCGGCATAACTGGCACTGCCCTAAGCCTACTGATCCGAGCAGAGCTRAGCCAGCCCGGCTCRCTCCTTGGTGACGACCAAATCTATAACGTAGTCGTGACAGCCCATGCATTCGTAATAATCTTTTTCATGGTGATGCCCGTTATGATCGGCGGGTTTGGCAACTGACTGGTGCCMATTATAATCGGCGCCCCGGACATAGCATTCCCACGAATAAATAACATAAGCTTCTGRCTCCTGCCCCCCTCCCTTCTACTACTGCTAGCCTCAGCAGGAATTGAAGCAGGGGCTGGWACAGGTTGAACCGTMTACCCRCCACTAGCAGCCAACYTAGCTCATGCTGGGGCCTCCGTGGACTTAGTAATTTTCTCGCTGCAYCTTGCTGGTGTGTCTTCTATCCTTGGGGCAATCAACTTTATTACCACRTGCATCAACATAAAATCCCCMTCAACAACACAATACACCACACCACTATTCGTTTGGTCCGTTTTAATCACAGCRAYCCTGCTACTACTAGCACTRCCGGTRCTTGCTGCAGGTATCACAATGCTTCTGACCGACCGAAACCTTAATACYACGTTYTTTGACCCGGCTGGAGGGGGCGACCCGATCTTGTACCAACACCTATTC

*Hemidactylus flaviviridis*

-------------------------------------------------------TTAGCCTCCTTATTCGGGCGGAGCTAAGTCAGCCGGGCTCGTTGCTTGGAGACGACCAAATCTACAACGTCGTGGTTACCGCCCATGCATTCGTAATAATCTTTTTTATAGTTATACCTATTATGATCGGCGGCTTCGGCAACTGACTAGTGCCATTAATAATTGGGGCACCAGACATGGCATTCCCCCGAATAAATAACATAAGCTTCTGGCTTCTGCCCCCTTCCCTTATCTTGCTGTTGGCCTCTGCCGGCGTTGAAGCCGGCGCAGGCACAGGCTGAACAGTCTATCCACCCCTGGCAGCCAACCTCGCACACGCCGGCGCATCTGTGGACCTTGTGATTTTTTCACTGCATCTTGCAGGTGTGTCATCCATTTTAGGCGCCATTAACTTTATTACAACATGTATCAACATGAAACCACCAGCCCTCTCGCAATACCACACCCCATTGTTTGTCTGGTCCGTACTAATTACAGCCGTACTACTATTGCTGGCACTCCCAGTGCTTGCCGCAGGGATTACTATGCTCCTGACAGATCGAAACATCAACACCACATTTTTTGACCCCGCCGGAGGCGGGGACCCAGTACTATACCAGCACCTATTC

*Hemidactylus forbesii*

ATCGGCACCTTATACCTACTGTTCGGTGCCTGAGCCGGCATAACCGGCACAGCCCTAAGTCTACTCATTCGAGCCGAGCTAAGCCAACCAGGAGCGCTGCTAGGAGACGATCAGATCTATAATGTAGTAGTAACTGCCCATGCATTTGTAATAATTTTTTTCATGGTTATGCCAATCATAATTGGCGGCTTCGGAAACTGACTGGTGCCCCTTATAATCGGCGCGCCAGACATAGCATTCCCCCGAATGAACAACATGAGCTTCTGACTTCTTCCGCCATCCCTATTACTATTACTCGCATCAGCTGGYATTGAAGCCGGTGCGGGCACTGGATGAACCGTATACCCCCCTCTAGCCGCCAACCTGGCTCATGCCGGAGCCTCTGTAGACCTGGTAATCTTCTCTCTCCACCTTGCTGGTGTGTCCTCCATCCTCGGGGCTATTAACTTTATCACCACGTGCATCAACATAAAATCCCCAACACTATCACAATACCACACCCCACTATTTGTTTGATCAGTATTAATCACCGCAGTGCTCCTACTTCTGGCCTTACCAGTTCTTGCCGCAGGAATCACTATGCTATTAACAGACCGAAACCTGAACACTACATTCTTTGATCCGGCTGGAGGAGGGGACCCAGTGCTCTATCAACATCTTTTT

*Hemidactylus granti*

ATTGGCACCCTATACCTACTATTTGGTGCCTGGGCCGGCATAACTGGCACTGCCTTAAGCCTCCTGATCCGAGCAGAGCTGAGCCAGCCCGGCTCACTCCTTGGYGACGACCAGATCTACAACGTAGTCGTGACAGCCCATGCATTTGTAATAATCTTTTTCATGGTGATGCCAGTCATGATCGGCGGGTTTGGTAACTGATTGGTGCCCATTATGATCGGMGCCCCAGACATAGCATTCCCACGAATAAACAACATAAGCTTCTGACTCCTTCCCCCCTCCCTTCTATTACTGCTGGCCTCAGCAGGAATTGAAGCAGGCGCTGGAACAGGCTGAACAGTCTACCCACCACTGGCAGCCAACTTAGCGCATGCCGGAGCCTCCGTGGATTTAGTGATTTTCTCACTGCATCTTGCTGGCGTGTCTTCCATTCTTGGCGCAATTAACTTTATTACCACGTGCATCAACATAAAATCCCCATCAATAACACAATACACCACACCCCTGTTCGTTTGGTCCGTTTTAATCACAGCAATTCTACTTCTACTAGCACTACCGGTACTTGCTGCAGGTATTACAATACTTCTGACCGACCGAAACCTTAACACCACGTTCTTTGACCCCGCTGGAGGGGGCGACCCCATTTTGTACCAACACTTATTC

*Hemidactylus homoeolepis*

ATCGGCACCCTDTAYCTYCTMTTTGGTGCCTGAGCCGGRNTRACYGGCACGGCCCTCAGCCTGCTCATCCGCGCCGAAYTAAGCCAGCCCGGDTCKCTAYTRGGMGACGACCARATCTATAATGTMGTRGTAACAGCCCACGCKTTCGTAATAATTTTCTTCATRGTAATGCCHATCATAATYGGCGGCTTYGGCAACTGACTTGTGCCCCTTATAATCGGSGCACCAGACATGGCRTTCCCCCGAATGAACAAYATRAGCTTCTGRYTGCTMCCCCCATCCTTACTACTAYTMCTMGCATCTGCTGGTGTAGAAGCHGGYGCCGGCACCGGCTGAACTGTCTAYCCGCCCCTAGCMGCCAACCTAGCACATGCCGGCGCCTCYGTKGACYTRGTAATTTTTTCMCTACACCTTGCCGGTGTMTCCTCCATTCTAGGKGCYATTAACTTTATCACCACRTGCATCAACATAAAAKCCCCAGCAATGWCACAATACCACACCCCCTTRTTTGTMTGRTCRGTAYTAATYACCGCMGTRCTVTTACTMCTAGCYCTGCCAGTKCTTGCYGCAGGCATCACCATGCTCTTRACMGATCGAAACCTRAACACCACMTTCTTCGACCCSGCTGGCGGNGGAGACCCAGTRYTATAYCARCACCTYTTC

*Hemidactylus inintellectus*

ATTGGCACCCTRTACYTACTRTTTGGYGCCTGRGCYGGYATARCKGGCRCCGCDCTRAGYMTYCTAATTCGAGCAGARCTRWGYCARSCYGGCWCACTMYTYGGVGACGAYCAARTYTACAACGTRRTBGTRACCGCYCAYGCMTTYGTRATAATCTTYTTCATRGTMATRCCARTCATAATTGGNGGGTTTGGYAACTGACTBGTRCCYCTTATRATYGGNGCCCCAGACATRGCATTCCCRCGAATRAACAACATRAGYTTCTGRYTRCTYCCHCCVTCYCTYCTTCTRCTGMTDGCCTCRGCCGGARTYGAAGCRGGDGCRGGAACAGGBTGAACAGTATACCCRCCMCTBGCAGCYAAYHTRGCCCAYGCTGGCGCMTCCGTRGAYCTRGTTATYTTCTCACTTCACCTTGCYGGYGTRTCYTCRATYYTHGGDGCAATYAAYTTTATTACCACRTGYATCAACATRAARTCCCCAACAGTRACACAATAYAACACCCCYYTRTTCGTVTGRTCYGTVCTAATCACRGCARTYCTRYTACTAYTAGCRCTYCCHGTVCTWGCCGCRGGRATCACAATACTCYTAACCGAYCGAAAYCTYAACACCACATTCTTYGACCCCGCYGGYGGNGGBGACCCHGTRYTRTACCANCAYCTRTTC

*Hemidactylus oxyrhinus*

ATCGGCACCCTGTATCTACTGTTCGGTGCCTGAGCCGGTATAACCGGCACAGCCCTAAGTCTCCTCATCCGAGCTGAATTAAGCCAGCCCGGRTCCCTGCTAGGAGATGATCAGATTTATAACGTAGTAGTGACTGCCCATGCRTTCGTAATRATCTTTTTCATAGTAATRCCAATCATAATTGGCGGCTTCGGCAACTGACTRGTACCCCTTATAATCGGCGCACCAGACATAGCATTCCCACGAATAAAYAACATGAGCTTTTGACTGCTTCCCCCCTCCCTACTACTACTGCTCGCATCTGCTGGAGTTGAAGCCGGCGCTGGCACCGGCTGAACCGTRTACCCCCCACTAGCCGCAAACCTRGCACATGCCGGAGCKTCTGTTGACCTGGTAATCTTCTCACTCCACCTTGCTGGCGTATCCTCCATCCTAGGGGCTATTAACTTTATCACCACATGCATCAACATAAAATCCCCAACACTRTCACAATAYCACACGCCACTGTTTGTATGATCAGTGYTAATCACCGCAGTACTACTRCTTCTAGCCYTGCCAGTTCTTGCCGCAGGCATCACCATRCTCTTAACAGATCGAAACCTCAACACCACATTCTTTGACCCTGCTGGAGGAGGAGACCCAGTGCTCTATCAACACCTTTTT

*Hemidactylus pumilio*

ATCGGCACCCTTTACYTAYTRTTYGGYGCCTGRGCCGGRATRACTGGNACSGCCCTRAGCCTCMTRATYCGYGCAGARCTHAGYCAGCCCGGRKCVCTMCTDGGVGAYGACCARRTYTACAACGTRGTMGTCACCGCYCATGCVTTCGTAATAATYTTCTTCATRGTYATRCCMATCATRATYGGVGGYTTTGGYAACTGRCTRRTCCCSCTYATAACRGGCGCCCCAGACATGGCRTTCCCRCGAATRAACAACATRAGYTTCTGRCTRCTCCCCCCVTCCCTCCTWCTYCTCCTYGCCTCBGCCGGMRTCGAAGCHGGYGCAGGVACKGGCTGAACRGTSTACCCRCCCCTCGCAGCCAACCTRGCRCACGCYGGCGCRTCAGTYGAYCTTGTAATYTTCTCVCTRCACCTRGCTGGYGTYTCATCMATCCTRGGGGCYATYAACTTTATCACYACATGYRYHAAYATAAAAGCCCCMRCMGYMTCACAATACAACACCCCMCTYTTCGTYTGRTCAGTVCTAATTACCGCAGTMCTGCTRCTRCTMGCACTKCCCGTVCTRGCMGCAGGCATCACYATRCTCYTRACYGATCGAAACYTAAACACAACMTTCTTYGAYCCTGCCGGRGGRGGVGACCCAGTRCTCTAYCARCACCTATTY

*Hemidactylus robustus* ATCGGCACCTTATACCTCCTGTTCGGCGCCTGAGCCGGAATAACCGGCACCGCACTCAGCCTCCTCATCCGCGCCGAACTTANCCAACCCGGCTCACTACTTGGCGATGACCAGATCTATAATGTCGTAGTRACCGCRCACGCATTCGTAATAATCTTCTTCATGGTAATRCCAATCATAATTGGTGGGTTCGGCAACTGACTGGTCCCACTMATGATCGGCGCGCCCGACATGGCATTCCCYCGTATAAACAACATGAGYTTCTGAYTRCTTCCYCCATCCCTACTACTACTACTGGCCTCTGCCGGAGTTGAAGCTGGCKCGGGAACAGGGTGAACCGTGTATCCGCCACTAGCAGCCAACCTTGCACATGCCGGCGCATCCGTAGACCTRGTTATTTTTTCACTACACCTTGCAGGTGTGTCATCAATTCTTGGGGCTATTAACTTCATCACCACGTGCATCAAYATAAAATCCCCAACCCTATCACAATATCACACCCCACTRTTTGTSTGATCAGTGTTAATTACCGCAGTACTACTAYTATTAGCCCTTCCAGTCCTCGCCGCAGGCATCACAATACTACTAACAGACCGYAACCTAAATACAACATTTTTTGATCCGGCTGGCGGAGGAGACCCAATTCTCTAYCAACACCTWTTC

*Mesalina balfouri*

ATYGGCACTYTATACCTYTTATTCGGTGCCTGAGCTGGRATGGTGGGAACAGCTTTAAGCCTAATTATTCGAACTGAGTTAAGCCAACCAGGTACCCTTCTCGGGGATGACCAGNTYTATAATGTTGTAGTTACRGCCCATGCCTTCGTAATAATCTTTTTCCTCGTAATACCAGTAATAATCGGCGGCTTTGGAAATTGACTRRTCCCCCTAATAATCGGGGCACCGGACATRGCATTTCCACGAATAAATAAYATAAGTTTTTGATTACTYCCMCCCTCTCTCCTCCTACTCCTATCATCCTCWATTATCGARGCCGGGGCCGGCACAGGTTGAACTGTTTACCCCCCACTGGCAGGTAATTTGGCCCACGCAGGGGCTTCCGTAGATTTAACAATCTTTTCATTACATTTGGCAGGYGTCTCCTCAATTCTTGGGGCAATTAACTTTATTACCACCTGYATTAACATRAAACCACCAAATATRACACAATACCAGACACCMTTATTTGTYTGATCCGTTTTAATTACAGCAGTACTTCTTCTACTTTCACTACCAGTATTAGCTGCAGGYATTACAATACTTTTGACAGATCGTAATCTAAACACMTCRTTCTTTGACCCVTCCGGGGGKGGRGACCCAATTCTATATCAACACCTRTTC

*Mesalina kuri*

ATCGGCACCTTATATCTCCTATTTGGTGCTTGAGCCGGAATGGTCGGAACGGCCCTAAGCTTRRTTATTCGAACTGAGCTCAGCCAACCAGGYACCCTTCTCGGTGATGATCAGGTCTACAATGTTGTAGTCACAGCCCATGCCTTCGTTATAATCTTTTTTCTTGTTATACCAGTAATAATCGGCGGCTTTGGAAATTGACTYGTGCCTCTAATAATTGGRGCCCCTGACATGGCATTCCCACGAATAAATAATATAAGCTTCTGAYTGCTCCCCCCATCRCTCCTTTTACTTTTAGCATCYTCTGCTGTTGAAGCAGGTGCTGGTACGGGTTGAACTGTTTACCCCCCACTGGCAGGAAACTTAGCCCACGCAGGAGCATCTGTAGAYTTAACAATCTTTTCGCTWCACTTAGCAGGCGTWTCCTCAATCCTTGGGGCAATTAACTTTATTACCACYTGCATCAATATAAAACCACCAAATATAACACAATATCAAACGCCCTTGTTCGTCTGATCCGTCTTAATTACAGCAGTACTTCTTCTACTTTCATTACCAGTATTAGCTGCAGGCATTACAATGCTTTTAACTGATCGTAACTTAAACACCTCCTTCTTTGATCCAGCCGGAGGCGGCGACCCAATTTTATACCAACACCTCTTC

*Myriopholis filiformis*

ATCGGCACCCTATACCTTTTATTCGGCGCATGATCAGGACTGATTGGCGCCACATTTAGCCTAATAATCCGCATAGAACTAGCTCAACCAGGAGCATTCTTAGAAAACGATCAAACCTATAACGTCATCGTAACCGCACACGCACTAATCATAATCTTCTTTATAGTCATACCAACACTTATCGGCGGCTTCGGAAACTGACTTATTCCAATAATAATTGGTGCACCAGATATAGCATTCCCTCGAATAAATAACATAAGCTTCTGACTTCTACCACCCTCACTAATACTACTATTGGCCTCCTCGTATATTGAAACAGGCGTTGGCACCGGCTGAACCATCTACCCACCCCTCTCAGGAAACCTAGCACACTCTGGACCATCAGTTGACCTAGCAATTTTCTCACTACACCTAGCAGGCGCCTCATCAATCCTAGGAGCAATCAACTTTATCACAACCTGCCTAAACATACTATCCACATCTATATCTATATATAACACATCTCTATTTGTCTGAGCCACCTTTATCACGGCTATCTTACTACTTATTGCCCTCCCAGTATTAGCAGCCGCAATCACCATACTCCTCACAGACCGCAATCTCAACACCTCATTTTTTGACCCAGCCGGAGGAGGAGACCCACTTCTATTTCAACACCTATTT

*Myriopholis macrurus*

ATTGGCACCCTTTACCTCATCTTGGGCGCATGATCAGGACTACTTGGAGCCACATTTAGCCTAATAATCCGYATAGAACTAGCCCARCCCGGAGCCTTYCTAGATAACGACCAAACCTACAACGTTGTAGTAACCGCGCACGCATTCATCATAATTTTCTTYATAGTTATACCAACACTAATTGGAGGCTTTGGAAACTGACTAATCCCAWTAATAATCGGCGCACCAGATATGGCCTTTCCACGAATAAACAATATAAGYTTCTGACTACTACCCCCATCCTTACTACTTCTACTCGCCTCCTCATACATCGAAACAGGCGTTGGAACRGGATGAACAGTATACCCCCCCCTYTCAGGAAATCTAGCACACTCAGGCCCATCAGTMGACCTAGCRATCTTTTCCCTTCACCTAGCAGGAGCCTCATCAATCCTAGGRGCAATCAACTTCATCACCACTTGTCTAAACATACTYACAACATCCATATCAATATACAACACATCCCTATTTGTATGAGCCACCTTCATYACAGCTATYCTAYTACTAATCGCACTYCCAGTACTAGCAGCCGCAATCACCATRCTYCTAACAGACCGCAAYCTTAACACCTCATTCTTCGACCCAGCCGGRGGAGGAGACCCAATCCTATTTCAACACTTATTC

*Myriopholis wilsoni* ATCGGCACCCTATACCTCCTRCTAGGCGCATGRTCTGGRYTAATTGGCGCCACATTTAGCCTAATAATCCGCATRGARCTRGCCCAACCWGGMGCCTTMTTAGAAAATGACCAAACATACAAYGTYATTGTAACAGCACAYGCATTCATCATAATCTTCTTCATRGTCATACCAACRCTYATCGGRGGRTTTGGRAACTGACTAATTCCAATAATAATTGGCGCMCCAGACATAGCCTTTCCACGAATAAACAAYATAAGCTTCTGATTATTACCCCCATCTYTACTTCTACTMCTAGCCTCCTCRTAYATYGAAACTGGCGTAGGTACGGGCTGAACCGTCTACCCACCRCTATCAGGAAAYCTAGCACACTCTGGCCCATCAGTAGACCTAGCAATTTTTTCMCTCCAYCTAGCAGGCGCATCCTCAATCYTAGGHGCAATTAAYTTTATCACCACATGCCTRAACATRCTATCAACATCCATATCAATATACAACACCTCRYTATTCGTATGAGCYACYTTYATTACAGCCATCTTACTACTAATCGCCCTACCAGTACTAGCAGCCGCAATYACCATACTMCTCACAGACCGCAATTTAAAYACCTCRTTTTTYGACCCRGCMGGAGGRGGRGAYCCCCTATTGTTTCARCATCTATTT

*Pachycalamus brevis* ATCGGCACATTATATCTTCTATTCGGCGCCTGAGCCGGTATGGCCGGCACCGCACTTAGCCTGCTTATTCGCGCTGAACTCGGCCAGCCCGGTGCCCTCTTAGGAGACGACCAAATTTACAATGTCGTCGTTACAGCTCACGCATTCGTCATAATCTTTTTCATGGTAATGCCGATCATGATCGGCGGCTTCGGAAACTGACTGGTACCCCTCATGATCGGTGCACCAGACATGGCCTTTCCGCGCATAAACAATATGAGCTTTTGACTTCTACCCCCATCACTACTCTTGTTACTGGCCTCTGCCGGCGTTGAAGCCGGCGCAGGCACGGGCTGAACCGTTTACCCCCCATTAGCCGGTAATTTAGCACACGCCGGGGCCTCAGTGGATCTAACAATTTTCTCACTACATCTAGCAGGGGTGTCCTCTATTCTTGGCGCAATTAATTTTATTACCACCTGCATTAACATAAAACCTCCGCGTATAACCCAATACCAAACACCCCTCTTTGTCTGATCGGTCATAATTACAGCAGTACTTTTACTACTCTCCCTACCAGTTCTGGCTGCGGGCATCACAATACTGCTCACAGATCGCAATCTAAATACAACCTTCTTTGACCCCTCCGGAGGCGGCGACCCAATTCTCTATCAACATTTATTC

*Pristurus abdelkuri*

ACCCTGTATCTCATYTTTGGTGCCTGGGCAGGAATAGTTGGCACCGCCCTCAGCCTGCTAATCCGTGCCGAGCTAAGCCAGCCCGGAGCCCTGCTGGGCGACGATCAGATCTATAACGTCATCGTCACCGCCCATGCATTCGTCATAATCTTTTTCATGGTTATGCCCATTATAATCGGCGGCTTTGGCAACTGACTCGTGCCCCTGATAATCGGCGCCCCCGACATAGCATTTCCGCGCATGAACAACATAAGCTTCTGACTGCTGCCCCCCTCACTACTACTACTGCTCTCCTCCTCCGCAATCGAGGCCGGCGCAGGAACGGGCTGAACAGTGTATCCCCCCCTAGCCTCCAACCTCGCACACGCGGGCATGTCCGTAGACTTGACAATCTTCTCACTTCACCTTGCCGGCGTATCATCTATCCTTGGCGCTATCAATTTTATCACCACCTGCGCCAACATAAAAGCACCTGCAATAACCATGTATAATATGCCCCTATTTGTCTGATCCGTATTAATCACCGCCGTGCTCCTCCTCCTATCCCTRCCCGTCCTCGCAGCAGGCATTACAATGCTRCTAACTGACCGCAATCTRAACACCACCTTCTTTGACCCTGCGGGAGGCGGCGAYCCAATCCTRTACCAACA-------

*Pristurus guichardi*

------ACCCTTTATCTCCTCTTCGGTGCCTGAGCYGGCATAGTCGGCACCGCCTTGAGTYTATTAATCCGTGCCGAAYTAAGCCAACCCGGGGCRCTCTTARGCGATGATCAAGTCTACAACGTTATTGTAACAGCCCACGCGTTCGTTATAATTTTTTTTATGGTCATACCAGTYATAATCGGCGGGTTTGGGAACTGACTTGTTCCACTGATATTAGGTGCCCCCGACATAGCATTTCCACGAATAAACAATATAAGTTTCTGGCTCTTACCCCCCTCCCTTCTCCTACTGCTTGCTTCATCCGCCGTCGAGGCTGGTGCTGGAACAGGCTGAACAGTATACCCCCCCCTTGCATCCAACTACGCTCACGCAGGTATATCTGTAGACCTGACTATTTTTTCATTACACCTTGCCGGTGTATCRTCCATCCTAGGGGCTATCAACTTTATTACTACCTGCGCTAACATAAAAGCCCCRGCAATAACCATGTATAATATGCCAYTMTTYGTGTGATCTGTTTTAATTACTGCCGTGCTACTCTTRTTATCCCTACCCGTTCTCGCAGCAGGAATTACCATGCTCCTCACAGATCGAAATCTTAATACCACCTTCTTTGACCCYGCCGGAGGGGGTGACCCTATCCTATACCAACACC-----

*Pristurus insignis*

ATCGGCACYCTYTAYCTYMTCTTCGGTGCCTGRGCTGGTATRGTYGGHACYGCCCTWAGYYTRCTAATYCGYGCTGAGCTVAGCCAGCCCGGRRCRCTKYTAGGYGAYGAYCARGTATATAATGTGATTGTCACTGCYCACGCSTTTGTWATAATYTTCTTCATGGTYATACCHATYATAATTGGKGGCTTYGGCAAYTGATTAGTRCCTTTRATAATTGGTGCCCCTGAYATAGCTTTYCCACGAATAAAYAAYATRAGCTTCTGRCTYCTACCVCCMTCRYTKYTACTWCTYCTTGCTTCMTCYRCAGTSGARGCMGGVRCCGGCACYGGVTGRACYGTYTACCCACCCCTCKCATCAAAYTAYGCMCATGCAGGKATRTCYGTAGAYYTRACYATTTTYTCYYTMCAYCTTGCCGGTGTRTCCTCTATYYTAGGRGCWATTAATTTCATTACYACBTGCGCYAAYATAAAAGCRCCYGCAATRTCYATVCAYAAYATRCCMCTTTTYGTYTGATCYGTMTTAGTMACYGCCRTYYTRTTACTCYTRTCYCTRCCRGTBYTMGCYGCRGGNATYACVATRCTYCTYACWGATCGRAACCTWAAYACYACMTTCTTCGACCCKGCVGGRGGYGGBGACCCTATYTTRTACCARCAYYTRTTT

*Pristurus insignoides*

ATCGGCACCCTTTATCTCRTCTTTGGYGCCTGAGCTGGTATAGTTGGTACCGCCTTAAGTTTACTAATTCGCGCTGAACTAAGCCAGCCGGGGACCCTTTTGGGTGATGACCAAGTGTACAATGTGATTGTTACCGCTCACGCCTTTGTAATAATTTTTTTTATGGTCATACCTATTATAATTGGCGGCTTTGGAAATTGGTTGGTGCCCTTAATAATTGGTGCCCCTGACATAGCCTTCCCACGGATAAACAATATAAGCTTTTGGTTGCTCCCCCCCTCATTACTGTTACTCCTCGCTTCCTCCGCAGTAGAAGCTGGTGCGGGCACAGGTTGAACCGTGTACCCACCCCTCGCATCAAACTACGCCCATGCAGGTATATCCGTAGATCTRACTATTTTCTCGTTACATCTTGCGGGTGTCTCCTCCATCCTGGGGGCCATTAACTTCATCACTACCTGCGCTAACATAAARGCACCMGCAATATCTATACATAACATACCCCTTTTCGTATGGTCTGTTCTAATCACTGCCGTACTACTACTCTTATCCCTCCCAGTCTTAGCTGCAGGYATTACGATACTTTTAACTGAYCGAAACTTRAATACTACCTTCTTTGACCCTGCAGGCGGTGGTGACCCYATTTTGTACCAACACCTGTTC

*Pristurus obsti*

-----CACCCTTTAYCTTCTCTTTGGTGCCTGAGCRGGYATAGTAGGCACCGCCTTAAGTTTACTAATCCGTGCCGAATTGAGCCAGCCTGGGGCGCTCCTAGGCGACGATCAAGTTTATAACGTAATTGTAACGGCTCACGCCTTTGTTATAATTTTTTTTATGGTTATACCAGTTATAATTGGCGGCTTTGGRAATTGACTTGTTCCATTGATACTCGGTGCCCCCGATATAGCATTCCCACGAATAAACAACATAAGTTTCTGGCTCCTGCCTCCCTCCTTACTCYTATTACTAGCTTCMTCCGCCGTTGAGGCTGGTGCCGGGACAGGCTGAACGGTATATCCCCCTCTTGCATCCAATTATGCTCATGCAGGCATATCTGTAGATCTGACTATTTTTTCATTGCACCTAGCAGGTGTATCTTCCATCTTAGGRGCTATTAACTTTATTACTACTTGTGCTAACATGAAAGCCCCGGCAATAACCATGTATAACATACCACTCTTTGTATGATCTGTCTTAATCACTGCCGTACTGCTCTTGCTATCCCTACCCGTCCTCGCAGCAGGAATTACCATGCTCCTTACGGATCGAAACCTAAATACCACTTTTTTTGAYCCTGCGGGCGGAGGTGAYCCTATCCTATACCAACA-------

*Pristurus samhaensis*

------------------------------TGAGCTGGCATAGTTGGCACGGCCCTAAGCTTGTTAATCCGCGCAGAGCTAAGCCAACCCGGCGCACTACTAGGCGATGATCAAGTTTATAATGTAATTGTTACAGCCCATGCCTTTGTTATAATTTTCTTTATGGTCATACCAATTATGATTGGTGGATTTGGTAATTGACTCGTTCCACTAATACTAGGTGCACCGGATATAGCCTTCCCACGAATAAACAATATAAGTTTCTGGCTTCTGCCCCCCTCCCTGCTCCTCTTACTTGCCTCATCCGCTGTCGAAGCCGGTGCCGGCACAGGCTGAACCGTTTACCCCCCTCTTGCATCCAATTACGCGCATGCAGGCATATCCGTAGACCTAACTATTTTCTCACTACATCTAGCCGGTGTATCCTCTATTYTAGGCGCTATCAATTTTATCACTACCTGTGCTAATATAAAAGCCCCGGCAATAACTATATACAACATGCCTCTTTTTGTATGATCTGTTCTAATCACTGCCGTCCTTCTCCTCTTATCTTTACCTGTCCTTGCAGCRGGAATTACTATGCTCCTCACTGATCGCAATTTAAATACAACCTTCTTTGACCCTGCTGGTGGCGGTGA-------------------------

*Pristurus sokotranus* ATCGGCACCCTTTAYNTACTYTTTGGTGCCTGAGCTGGHATAGTTGGHACMGCCYTRAGYYTDNTAATCCGYGCDGAGYTMAGCCARCCMGGYRCACTDCTNGGYGAYGAYCAAGTTNAYAATGTHATTGTDACRGCCCATGCNTTYGTTATAATYTTYTTTATRGTHATRCCAATYATRATNGGBGGSTTTGGYAAYTGRCTYGTYCCHYTAATACTWGGBGCWCCKGATATRGCCTTNCCACGAATRAACAAYATRAGYTTCTGRCTYYTRCCYCCCTCBCTGCTHCTYYTACTTGCCTCNTCBGCTGTCGARGCBGGYGCYGGYACAGGYTGAACMGTYTAYCCYCCMCTTGCATCCAAYTAYGCNCATGCAGGYATATCYGTAGACCTRACTATTTTYTCAYTRCAYYTRGCYGGYGTRTCCTCTATTTTRGGHGCNATYAAYTTTATYACYACYTGTGCTAATATAAAAGCCCCRGCAATAACYRTATAYAAYATRCCTCTTTTYGTRTGRTCYGTTYTRATCACYGCTGTRCTTCTYYTMYTRTCYTTACCTGTYCTTGCRGCAGGAATTACTATRCTYCTYACHGATCGCAATTTAAAYACWACYTTCTTTGACCCYGCTGGYGGNGGYNAYCNAATYTTRTAYCARCACCTATTC

*Trachylepis cristinae* ATTGGCACCCTATACTTAATCTTTGGTGCTTGAGCAGGTATAGTCGGGACTGCTCTAAGCCTCCTAATCCGGGCTGAACTAAGCCAACCAGGGACCCTTCTAGGGGATGATCAGATTTATAACGTGATTGTCACGGCACATGCCTTTGTAATAATTTTCTTTATAGTAATACCCGTAATGATCGGCGGATTTGGAAACTGGCTTGTTCCCCTAATAATTGGAGCGCCTGACATAGCATTTCCACGAATAAACAACATAAGCTTCTGACTTCTACCACCCTCATTCCTCCTCTTATTAGCCTCATCTGGTGTAGAAGCTGGAGCAGGGACTGGTTGGACAGTGTACCCACCACTCGCCGGAAACCTGGCCCATGCAGGAGCCTCTGTTGACCTTACTATTTTTTCCCTTCATTTAGCAGGAGTGTCATCAATCCTCGGGGCTATTAATTTTATTACTACATGTATCAACATAAAACCCCCAACAATAACACAATACCAAACTCCTTTATTTGTCTGATCTGTACTAATTACAGCAGTACTATTACTCTTATCCCTGCCCGTACTGGCAGCTGGCATTACCATGCTCCTAACAGATCGAAACTTAAATACATCATTTTTTGATCCTGCAGGAGGAGGGGACCCAATCCTCTACCAGCACCTGTTC

*Trachylepis socotrana*

ATCGGCACCCTGTATTTGATCTTTGGTGCCTGAGCAGGAATARTCGGAACCGCCCTGAGCCTCCTAATTCGGGCCGAAYTRAGCCAACCCGGGRCTCTCTTGGGTGAYGACCAGATCTATAACGTCATTGTTACAGCCCACGCCTTTGTAATAATTTTCTTCATRGTAATGCCTGTSATAATCGGCGGGTTTGGGAATTGGCTTGTCCCGCTAATAATCGGCGCCCCTGATATGGCATTCCCCCGAATAAAYAACATGAGCTTTTGACTRCTCCCACCATCTTTCCTTCTAYTATTGGCCTCTTCGGGGGTGGAGGCCGGAGCTGGGACTGGYTGGACCGTGTACCCACCACTAGCCGGCAACCTRGCCCACGCCGGAGCCTCTGTAGACTTAACTATCTTTTCCCTTCATCTTGCAGGGGTGTCATCAATCCTTGGKGCCATTAACTTTATCACCACCTGYATTAAYATAAAACCTCCGACAATAACRCAATACCAAACCCCACTGTTTGTRTGATCAGTACTAATCACCGCTGTMCTACTACTACTATCTTTACCCGTCTTAGCAGCCGGAATCACCATRCTTCTYACAGACCGAAACCTAAATACATCATTTTTTGATCCAGCAGGCGGKGGAGACCCAATTTTATACCAACAYCTGTTC

*Xerotyphlops socotranus* ATCGGCACCCTATATTTACTATTCGGCGCCTGATCCGGTATCCTAGGAGCAGCCCTAAGCATAATAATCCGAATGGAACTATGCCAACCGGGCTCCCTCCTTGGAAATGATCAAATTTATAATGTAGTAGTTACAGCCCACGCATTCGTAATAATCTTTTTTATGGTTATACCAACAATAATCGGAGGCTTTGGAAACTGACTAGTCCCATTAATACTGGGGGCACCAGACATAGCATTCCCACGCATAAATAATATAAGCTTTTGACTTCTCCCCCCATCATTATTCTTACTACTAGCATCCTCATATGTAGAAGCTGGGGCTGGCACAGGATGAACTGTTTACCCCCCACTATCTGGTAACCTAACACACTCCGGCGCCTCAGTAGACCTAGCAATTTTTTCTCTCCATCTAGCTGGGATATCATCAATTCTTGGAGCAATTAACTTTATCACAACCTGCTTTAGCATAAAATCACCATCCATCACCCATTACAAATTTCCACTTTTCGTATGATCAGTACTAGCCACAGCAACTCTATTACTAATCGCACTGCCAGTGCTAGCAGCAGCTATCACTATACTACTAACTGATCGAAACATTAACACCTCCTTCTTTGACCCCTCGGGGGGAGGAGACCCAATCCTATTTCAACATCTATTC
