# Supplementary material for: Unexpectedly High Levels of Cryptic Diversity Uncovered by a Complete DNA Barcoding of Reptiles of the Socotra Archipelago
Source: PLoS One. 2016 Mar 1;11(3):e0149985. doi: 10.1371/journal.pone.0149985 (PMC4772999; doi:10.1371/journal.pone.0149985)

**Supporting Information**

**Unexpectedly high levels of cryptic diversity uncovered by a complete DNA barcoding of reptiles of the Socotra Archipelago**

**Raquel Vasconcelos, Santiago Montero-Mendieta,**

**Marc Simó-Riudalbas,**

**Roberto Sindaco,**

**Xavier Santos,**

**Mauro Fasola,**

**Gustavo Llorente**

**Edoardo Razzetti**

**Salvador Carranza**

**S1** **Fig. Species maps showing the localities and phylogenetic relationships of all Socotran reptiles.** A total of 380 specimens of included in this study. White dots represent bibliographic and new distribution records, and green stars sampled specimens. Black dots on trees indicate posterior probability values ≥ 0.95, and values next to the nodes Maximum Likelihood bootstraps ≥ 70%. Maps were drawn using DIVA-GIS v.7.5 (available at <http://www.diva-gis.org>). Photos reprinted [27] with permission from Edoardo Razzetti and Roberto Sindaco.

**
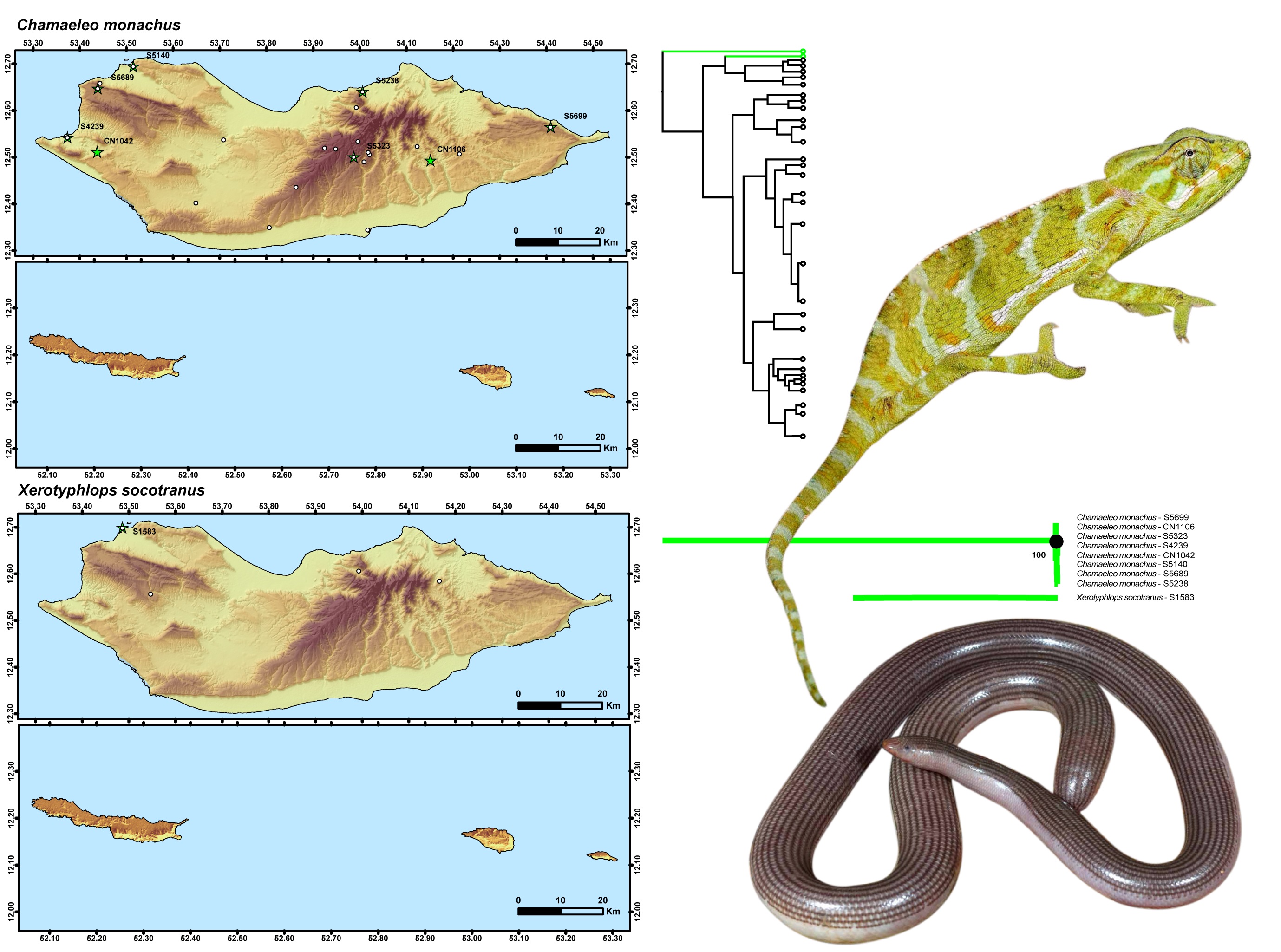
**

**
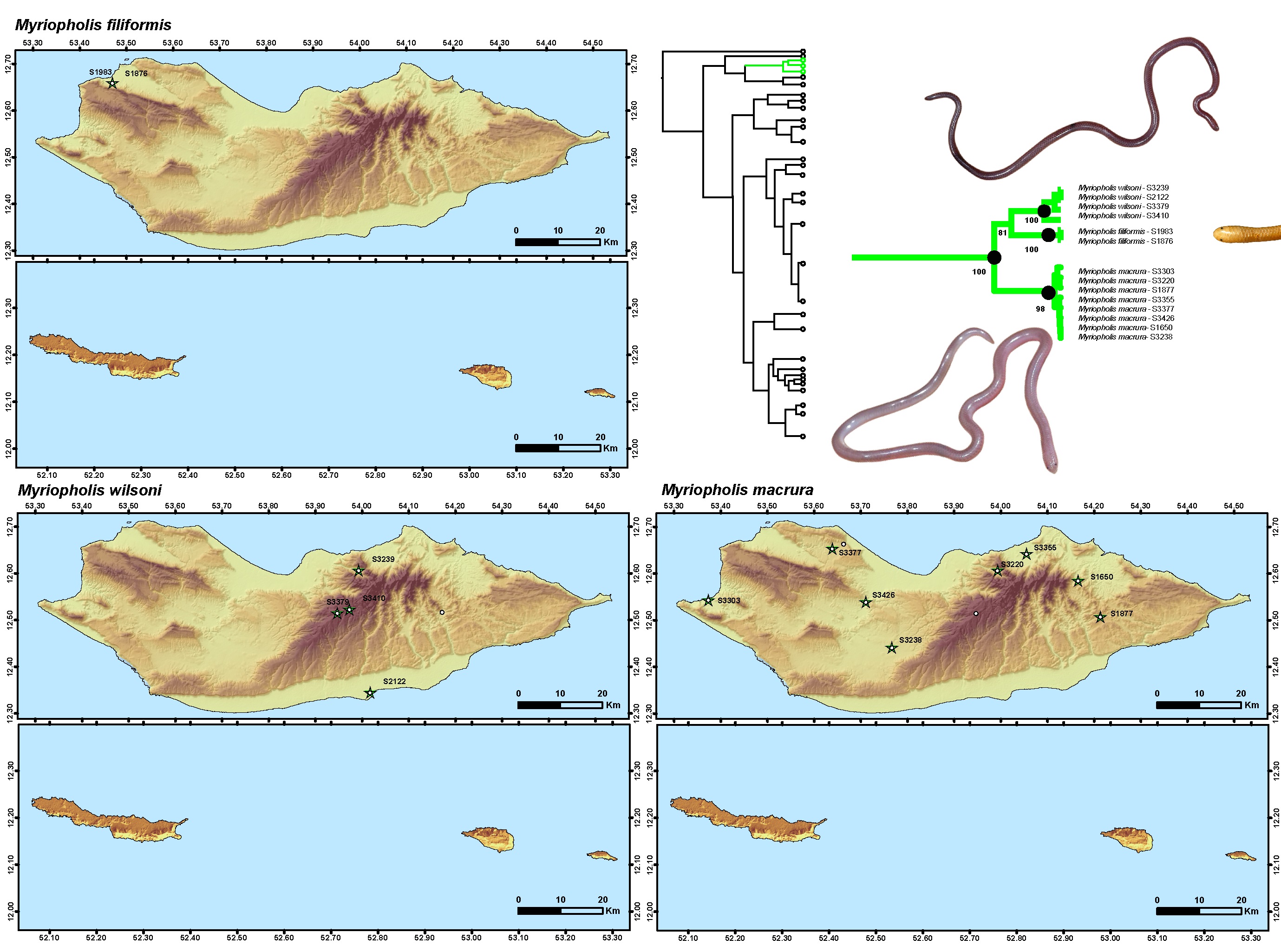
**

**
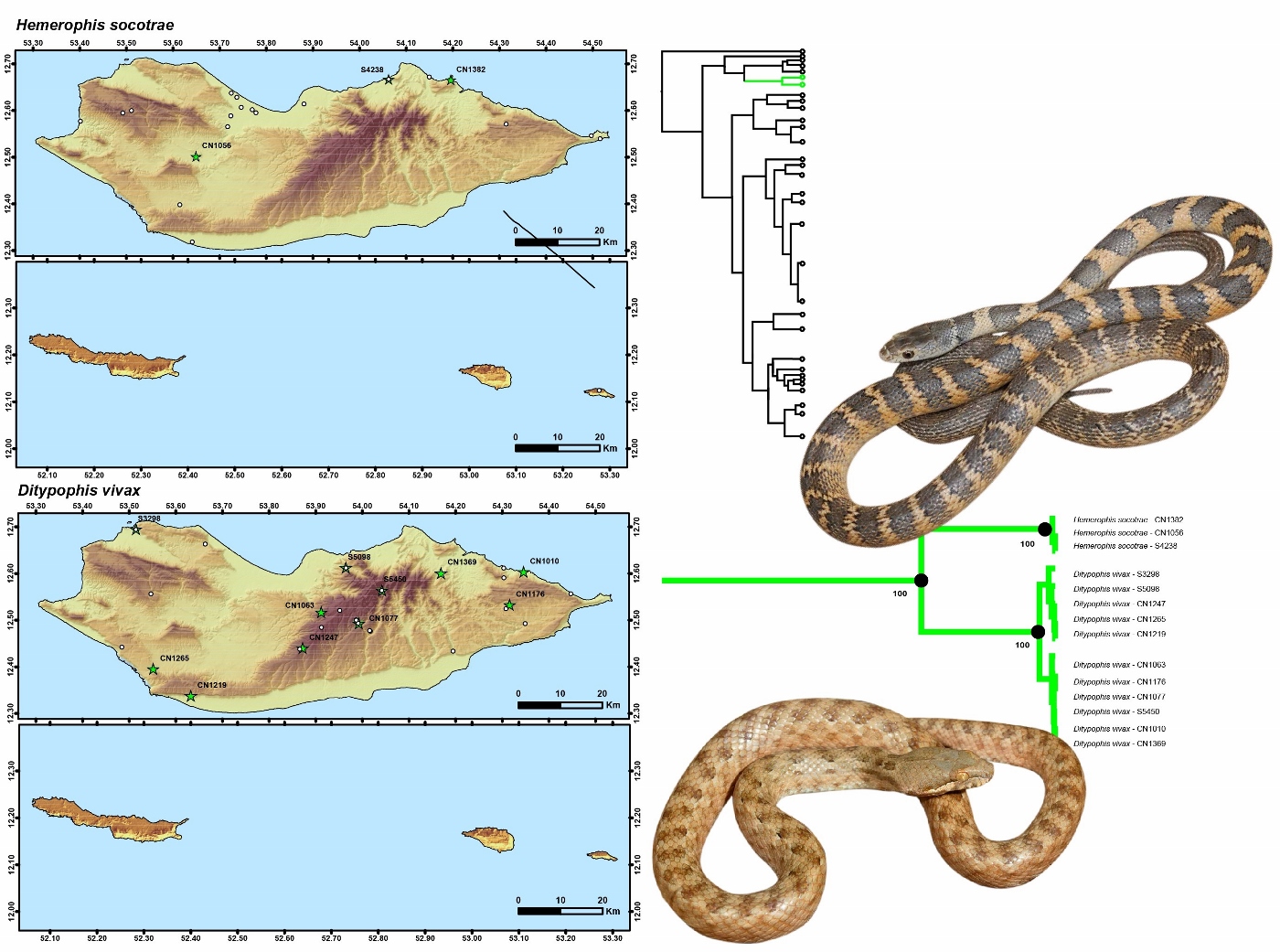
**


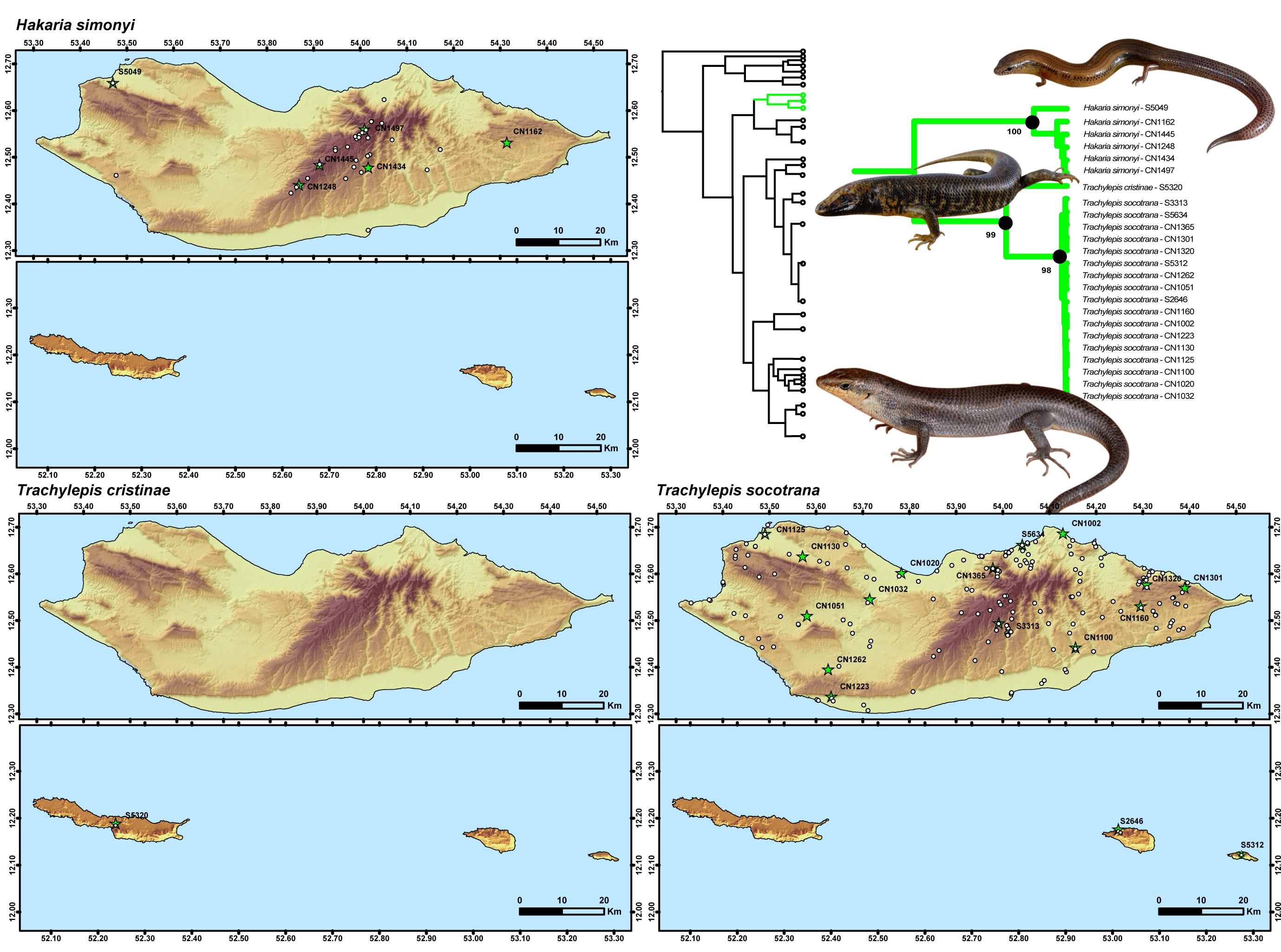


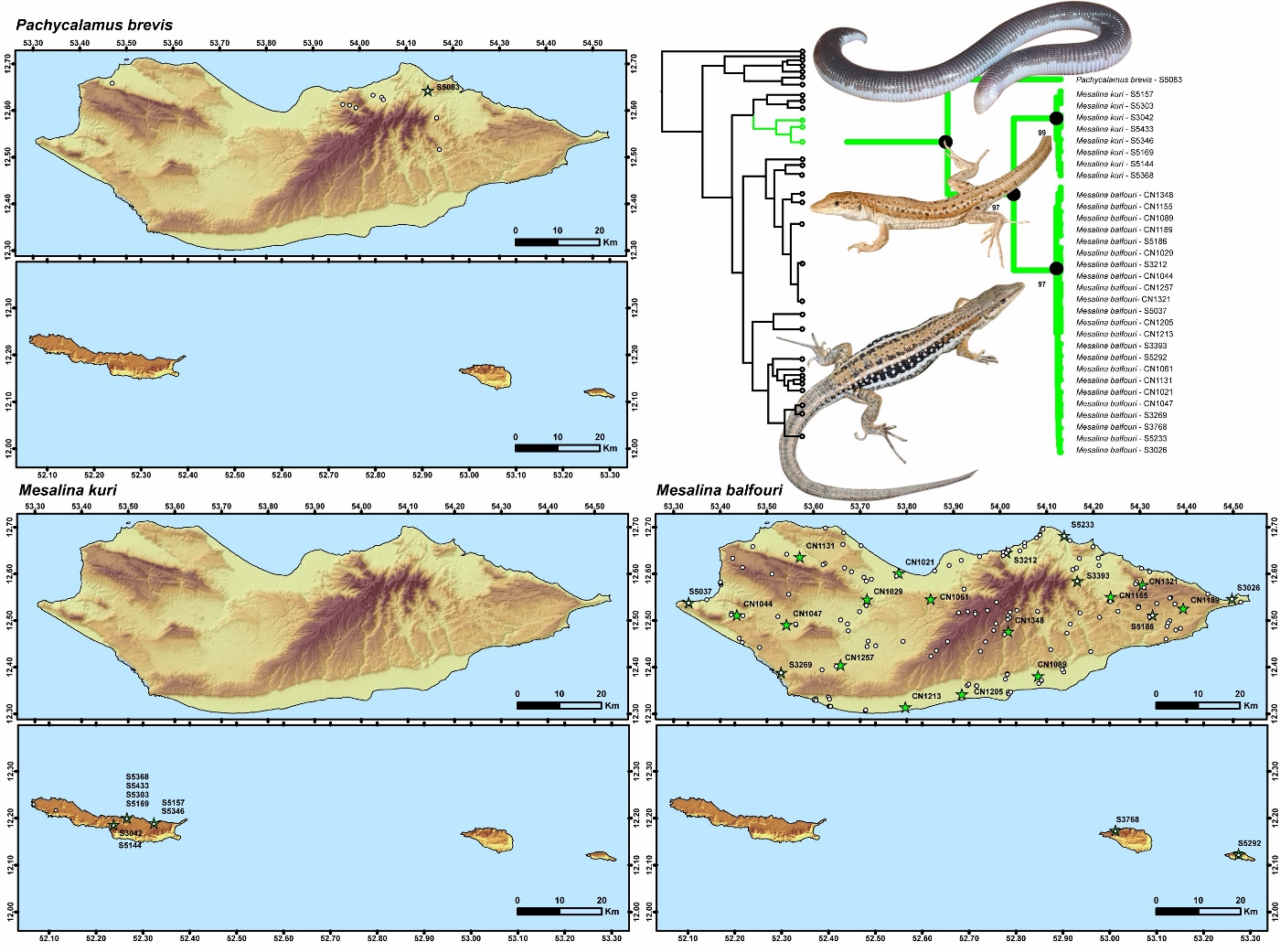


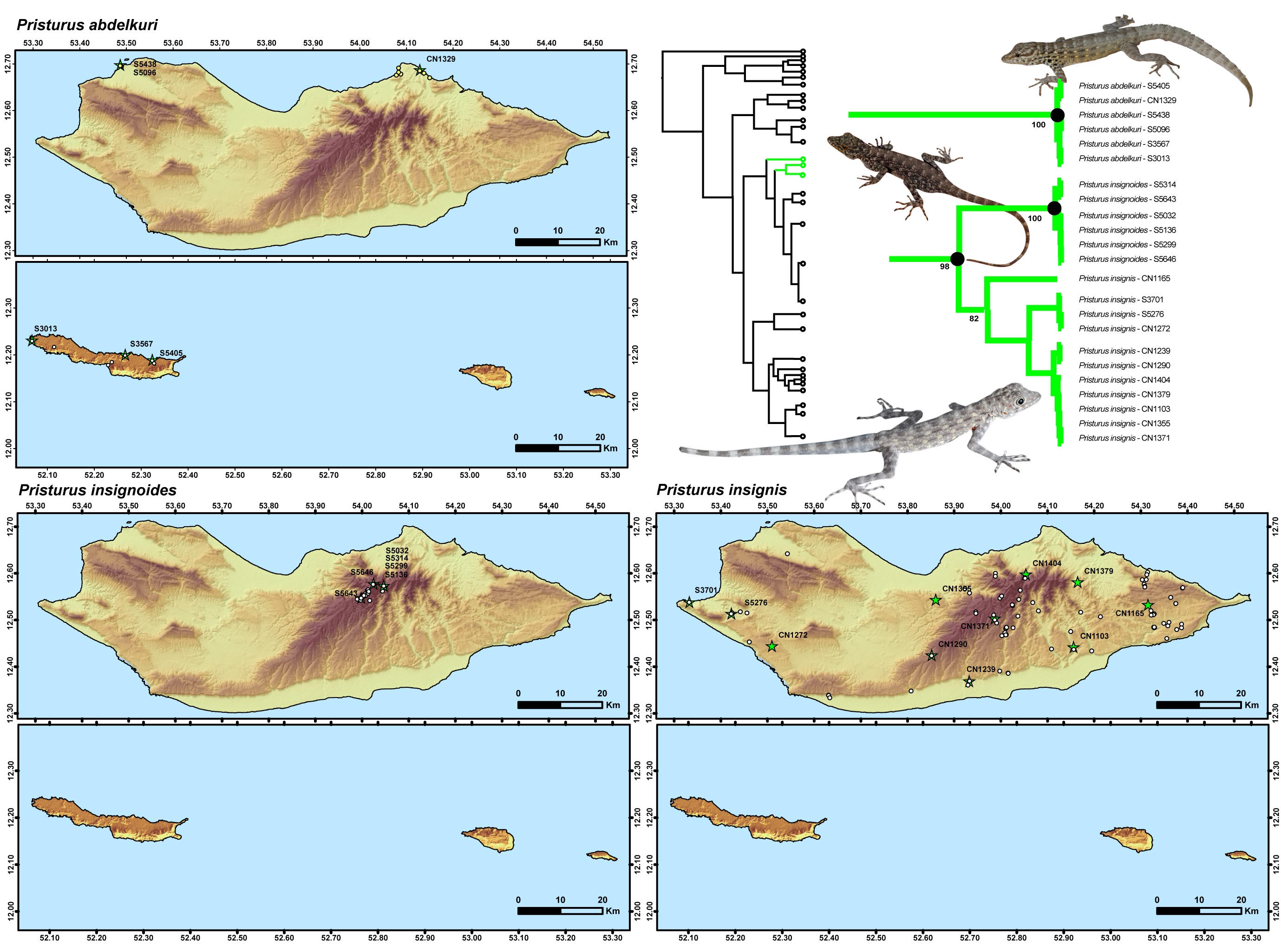


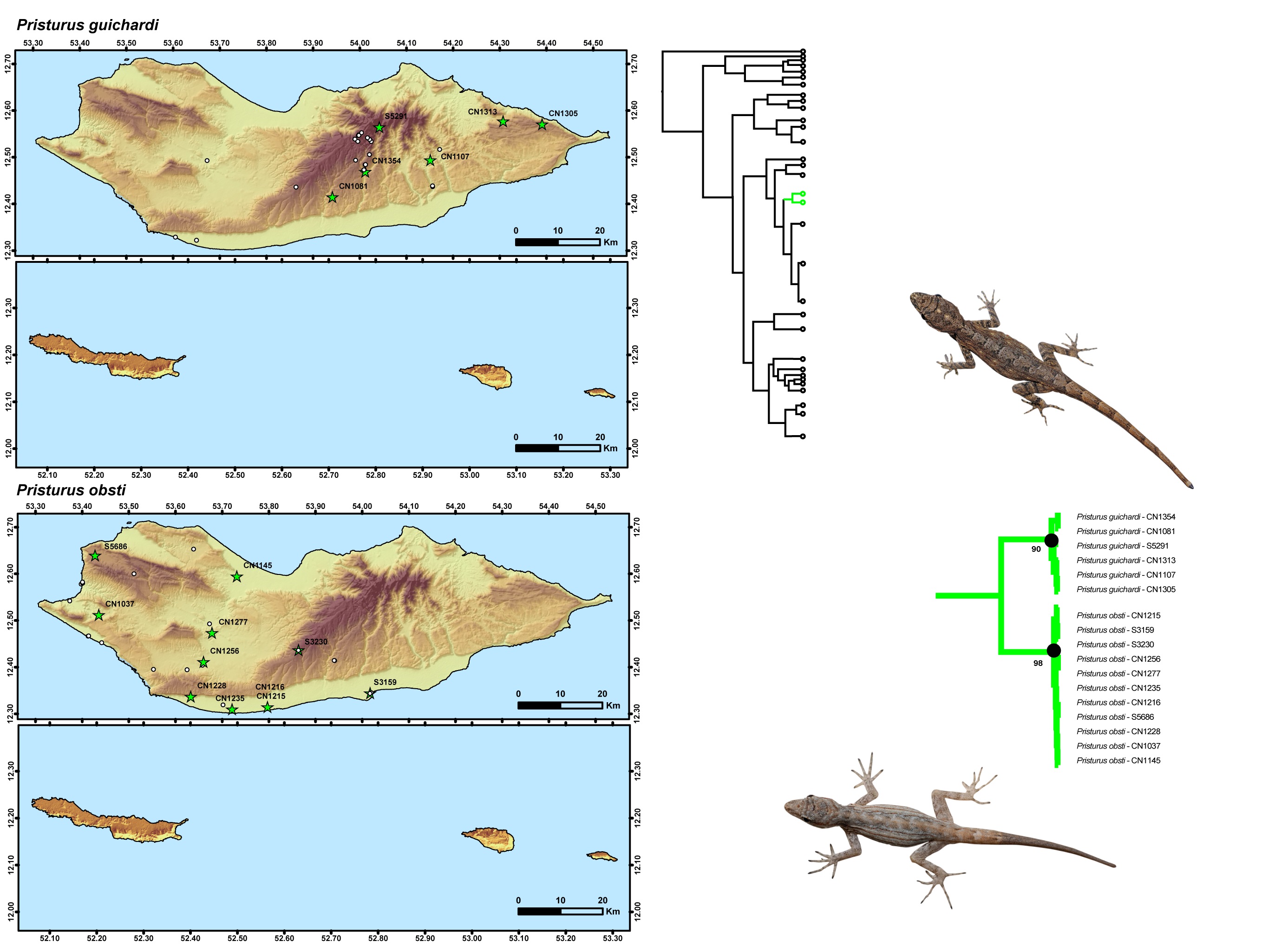


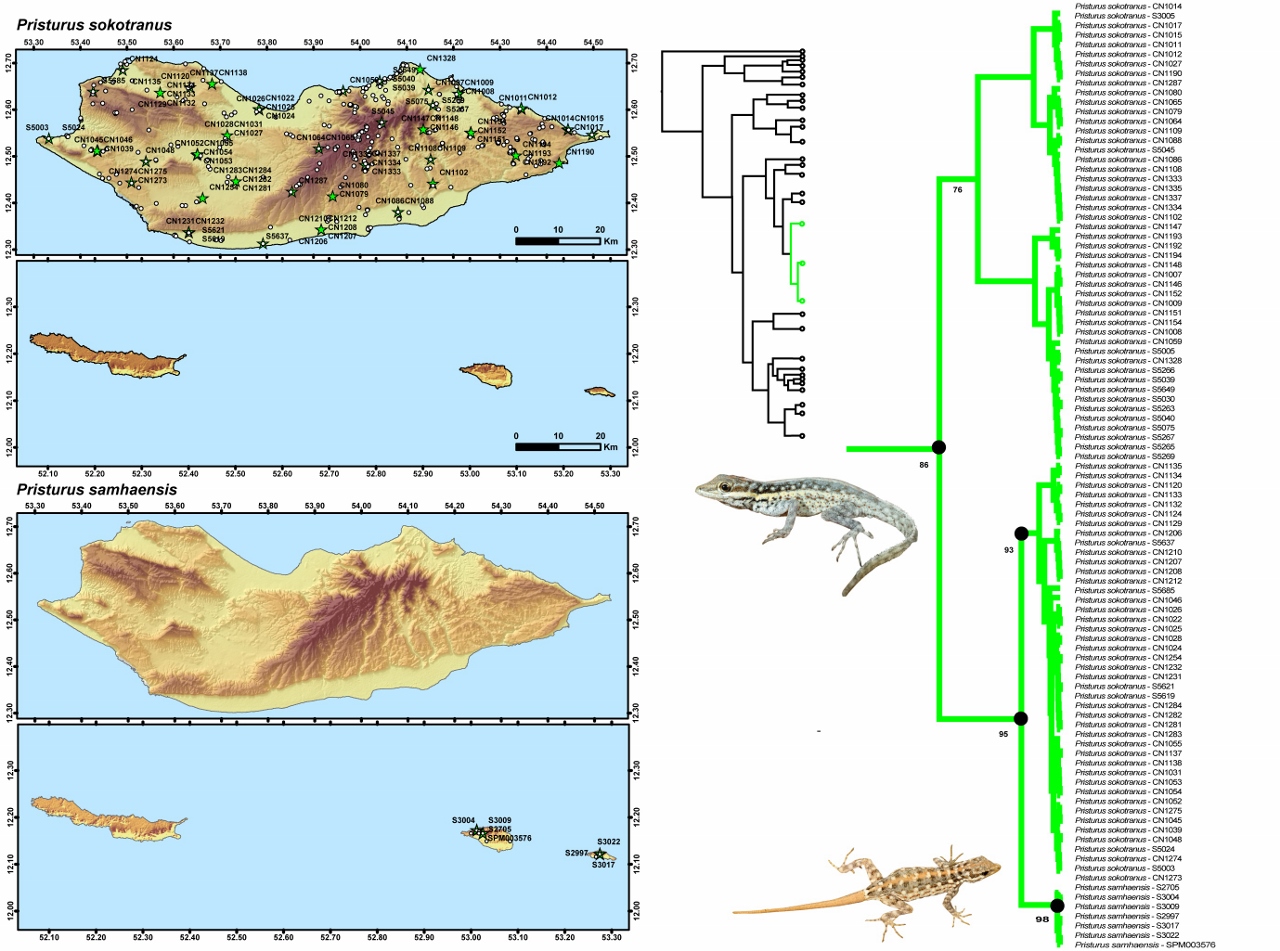


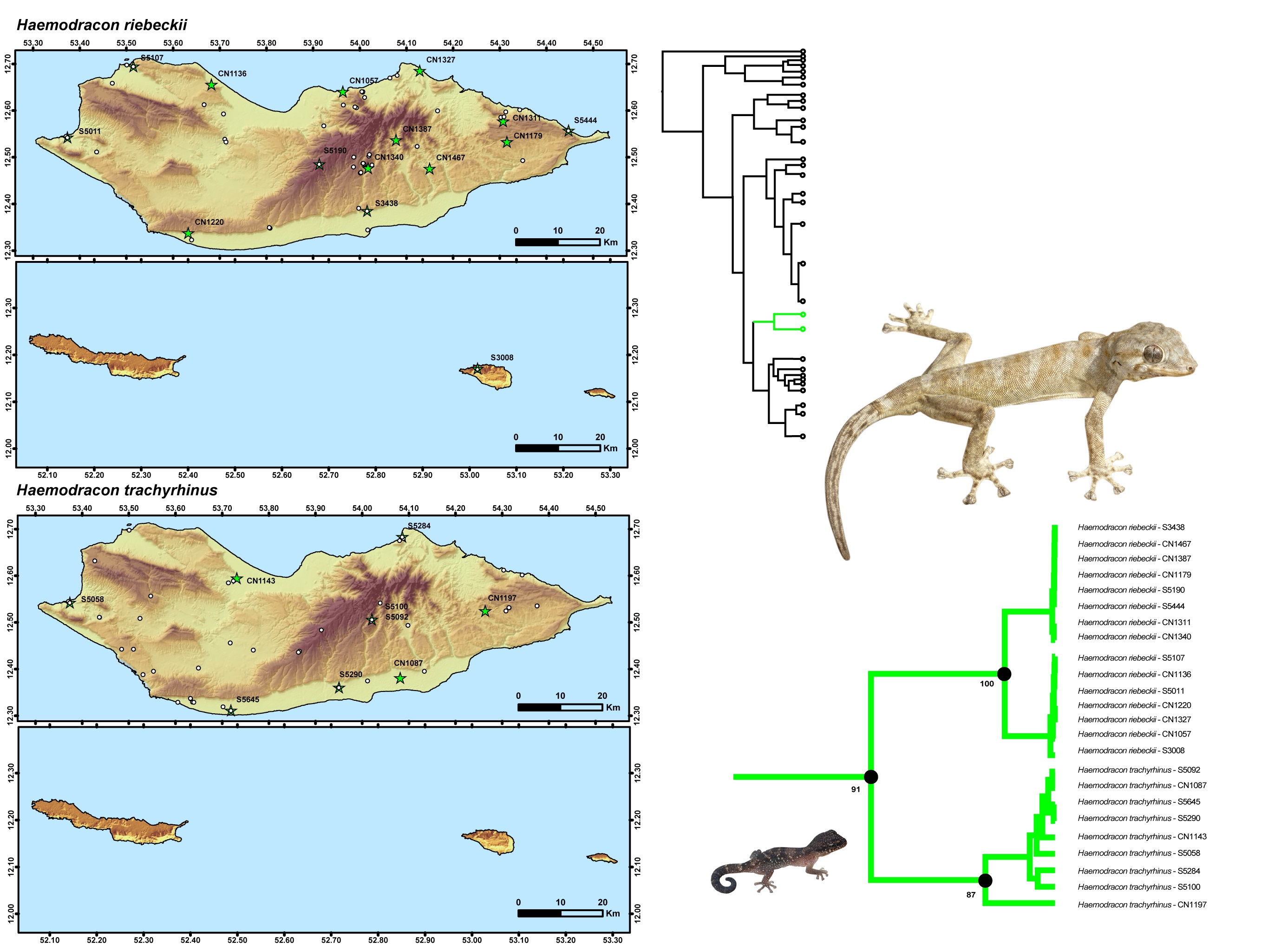


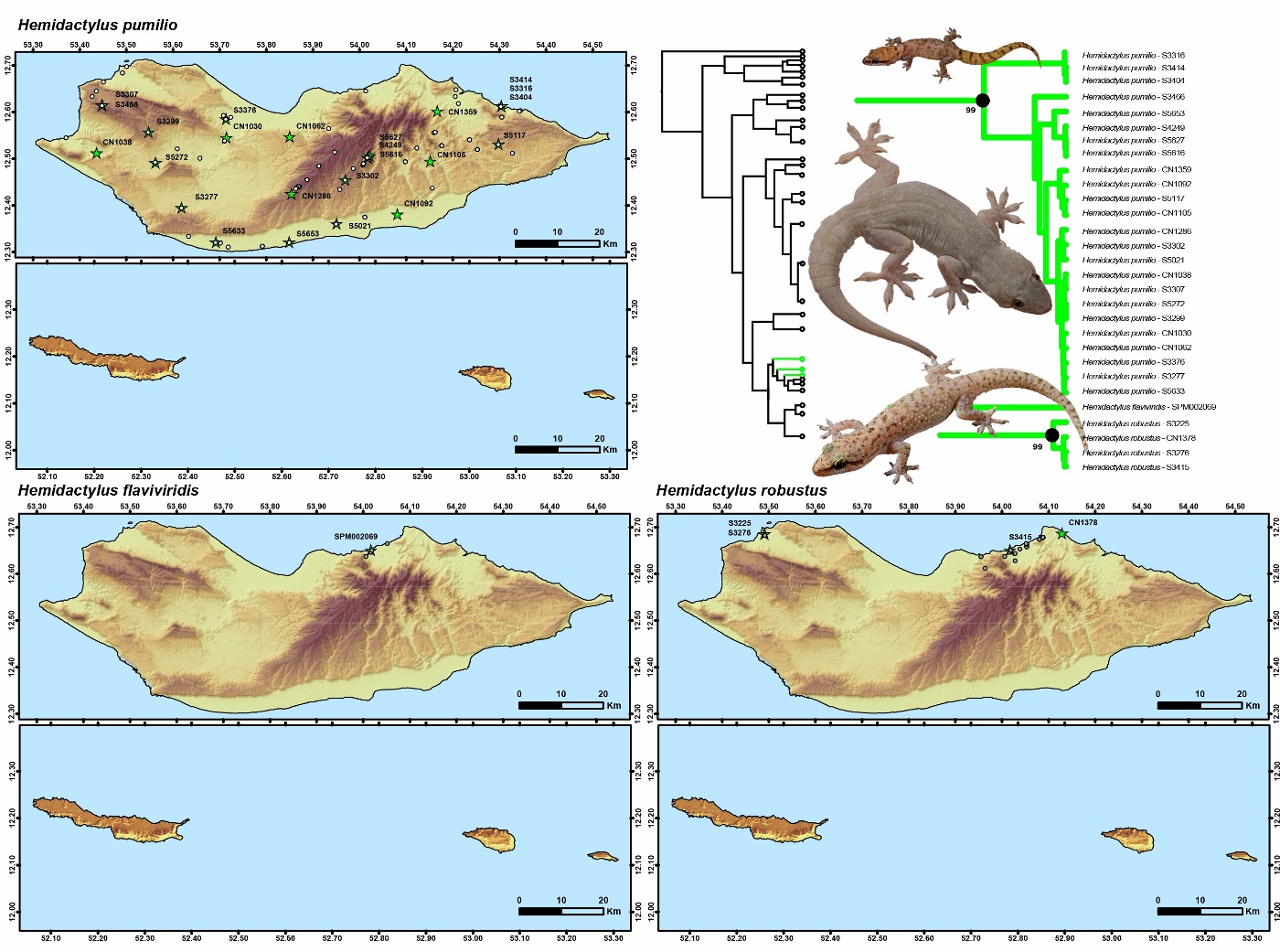


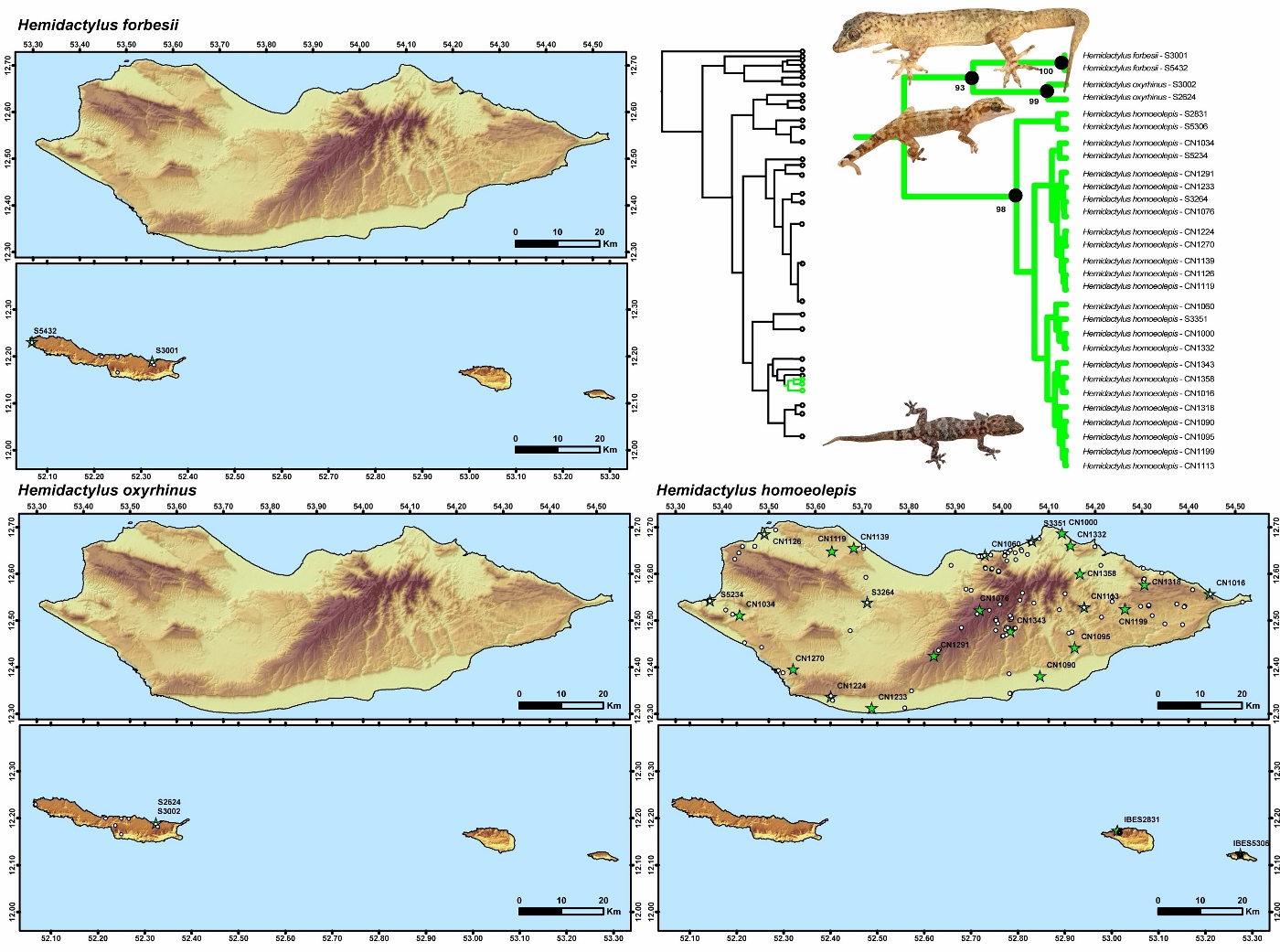


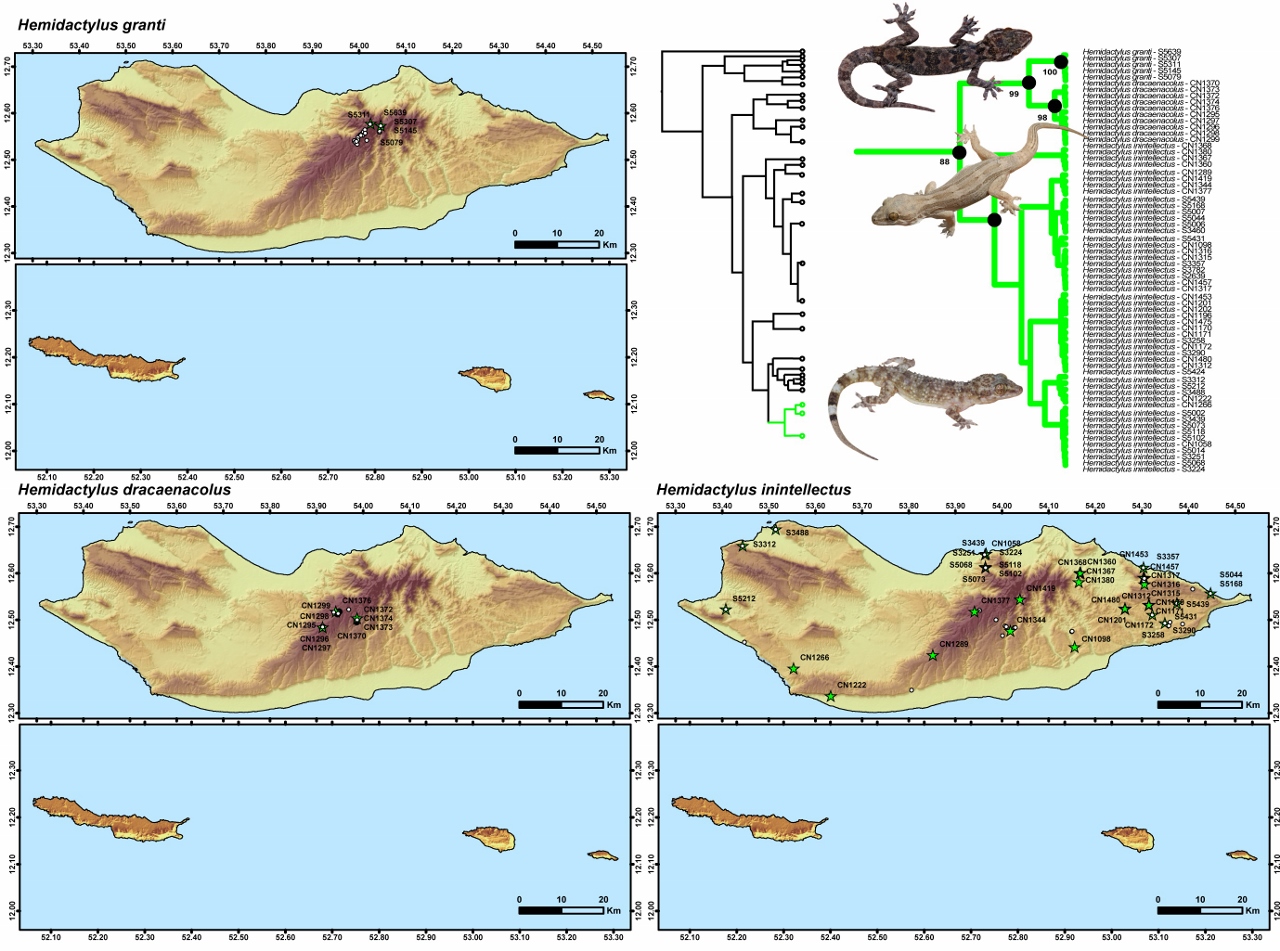

Supplement: S1 Fig — A total of 380 specimens of included in this study. White dots represent bibliographic and new distribution records, and green stars sampled specimens. Black dots on trees indicate posterior probability values ≥ 0.95, and values next to the nodes Maximum Likelihood bootstraps ≥ 70%. Maps were drawn using DIVA-GIS v.7.5 (available at http://www.diva-gis.org). Photos reprinted [27] with permission from Edoardo Razzetti and Roberto Sindaco. (DOCX) [file pone.0149985.s002.docx]
