# Supplementary material for: Unexpectedly High Levels of Cryptic Diversity Uncovered by a Complete DNA Barcoding of Reptiles of the Socotra Archipelago
Source: PLoS One. 2016 Mar 1;11(3):e0149985. doi: 10.1371/journal.pone.0149985 (PMC4772999; doi:10.1371/journal.pone.0149985)
Supplement: S2 Fig — Phylogenetic relationships based on COI gene. Bootstrap values >70% are shown next to the nodes. (DOCX) [file pone.0149985.s003.docx]

**Supporting Information**

**Unexpectedly high levels of cryptic diversity uncovered by a complete DNA barcoding of reptiles of the Socotra Archipelago**

**Raquel Vasconcelos, Santiago Montero-Mendieta,**

**Marc Simó-Riudalbas,**

**Roberto Sindaco,**

**Xavier Santos,**

**Mauro Fasola,**

**Gustavo Llorente**

**Edoardo Razzetti**

**Salvador Carranza**

**S2** **Fig. Maximum likelihood phylogenetic tree** **for all reptiles of the Socotra Archipelago**. Phylogenetic relationships based on COI gene. Bootstrap values >70% are shown next to the nodes.
