# Supplementary material for: Unexpectedly High Levels of Cryptic Diversity Uncovered by a Complete DNA Barcoding of Reptiles of the Socotra Archipelago
Source: PLoS One. 2016 Mar 1;11(3):e0149985. doi: 10.1371/journal.pone.0149985 (PMC4772999; doi:10.1371/journal.pone.0149985)
Supplement: S3 Fig — Bootstrap values ≥70% are shown next to the nodes. See Material and Methods for further details. (DOCX) [file pone.0149985.s004.docx]

**Supporting Information**

**Unexpectedly high levels of cryptic diversity uncovered by a complete DNA barcoding of reptiles of the Socotra Archipelago**

**Raquel Vasconcelos, Santiago Montero-Mendieta,**

**Marc Simó-Riudalbas,**

**Roberto Sindaco,**

**Xavier Santos,**

**Mauro Fasola,**

**Gustavo Llorente**

**Edoardo Razzetti**

**Salvador Carranza**

**S3 Fig.** **Neighbour-Joining tree for all reptiles of the Socotra Archipelago.** Bootstrap values ≥70% are shown next to the nodes. See Material and Methods for further details.
