# Supplementary material for: Unexpectedly High Levels of Cryptic Diversity Uncovered by a Complete DNA Barcoding of Reptiles of the Socotra Archipelago
Source: PLoS One. 2016 Mar 1;11(3):e0149985. doi: 10.1371/journal.pone.0149985 (PMC4772999; doi:10.1371/journal.pone.0149985)
Supplement: S1 Table — Taxonomic information and number of COI sequences amplified (N) of endemic and introduced reptile (*) species present (•) in the Socotra Archipelago. Question mark (?) stands for unchecked bibliographic records following recent work [27]. (DOCX) [file pone.0149985.s005.docx]

**Supporting Information**

**Unexpectedly high levels of cryptic diversity uncovered by a complete DNA barcoding of reptiles of the Socotra Archipelago**

**Raquel Vasconcelos, Santiago Montero-Mendieta,**

**Marc Simó-Riudalbas,**

**Roberto Sindaco,**

**Xavier Santos,**

**Mauro Fasola,**

**Gustavo Llorente**

**Edoardo Razzetti**

**Salvador Carranza**

**S1 Table.** **Details of the samples.** Taxonomic information and number of COI sequences amplified (N) of endemic and introduced reptile (*) species present (•) in the Socotra Archipelago. Question mark (?) stands for unchecked bibliographic records following recent work [27].

| **Squamata subgroups** | **Family** | **Species** | **N** | **Socotra** | **Abd al Kuri** | **Samha** | **Darsa** |
| --- | --- | --- | --- | --- | --- | --- | --- |
| **Infraorder**  **Iguania** | Chamaeleonidae | *Chamaeleo monachus* | 8 | • | - | - | - |
| **Suborder**  **Serpentes** | Typhlopidae | *Xerotyphlops socotranus* | 1 | • | - | - | - |
|  | Leptotyphlopidae | *Myriopholis filiformis* | 2 | • | - | - | - |
|  |  | *Myriopholis wilsoni* | 4 | • | - | - | - |
|  |  | *Myriopholis macrura* | 8 | • | - | - | - |
|  | Colubridae | *Hemerophis socotrae* | 3 | • | - | ? | • |
|  | Lamprophiidae | *Ditypophis vivax* | 11 | • | - | - | - |
| **Suborder**  **Scincoidea** | Scincidae | *Hakaria simonyi* | 6 | • | - | - | - |
|  |  | *Trachylepis cristinae* | 1 | - | • | - | - |
|  |  | *Trachylepis socotrana* | 17 | • | - | • | • |
| **Suborder Lacertibaenia** | Trogonophiidae | *Pachycalamus brevis* | 1 | • | - | - | - |
|  | Lacertidae | *Mesalina kuri* | 8 | - | • | - | - |
|  |  | *Mesalina balfouri* | 23 | • | - | • | • |
| **Infraorder**  **Gekkota** | Sphaerodactylidae | *Pristurus abdelkuri* | 6 | * | • | - | - |
|  |  | *Pristurus insignoides* | 6 | • | - | - | - |
|  |  | *Pristurus insignis* | 11 | • | - | - | - |
|  |  | *Pristurus guichardi* | 6 | • | - | - | - |
|  |  | *Pristurus obsti* | 11 | • | - | - | - |
|  |  | *Pristurus sokotranus* | 92 | • | - | - | - |
|  |  | *Pristurus samhaensis* | 7 | - | - | • | • |
|  | Phyllodactylidae | *Haemodracon trachyrhinus* | 9 | • | - | - | - |
|  |  | *Haemodracon riebeckii* | 15 | • | - | • | - |
|  | Gekkonidae | *Hemidactylus pumilio* | 24 | • | - | - | - |
|  |  | *Hemidactylus flaviviridis** | 1 | • | - | - | - |
|  |  | *Hemidactylus robustus** | 4 | • | - | • | - |
|  |  | *Hemidactylus forbesi* | 2 | - | • | - | - |
|  |  | *Hemidactylus oxyrhinus* | 2 | - | • | - | - |
|  |  | *Hemidactylus homoeolepis* | 25 | • | - | • | • |
|  |  | *Hemidactylus granti* | 5 | • | - | - | - |
|  |  | *Hemidactylus dracaenacolus* | 10 | • | - | - | - |
|  |  | *Hemidactylus inintellectus* | 51 | • | - | - | - |
