# Supplementary material for: Unexpectedly High Levels of Cryptic Diversity Uncovered by a Complete DNA Barcoding of Reptiles of the Socotra Archipelago
Source: PLoS One. 2016 Mar 1;11(3):e0149985. doi: 10.1371/journal.pone.0149985 (PMC4772999; doi:10.1371/journal.pone.0149985)
Supplement: S2 Table — (DOCX) [file pone.0149985.s006.docx]

**Supporting Information**

**Unexpectedly high levels of cryptic diversity uncovered by a complete DNA barcoding of reptiles of the Socotra Archipelago**

**Raquel Vasconcelos, Santiago Montero-Mendieta,**

**Marc Simó-Riudalbas,**

**Roberto Sindaco,**

**Xavier Santos,**

**Mauro Fasola,**

**Gustavo Llorente**

**Edoardo Razzetti**

**Salvador Carranza**

**Table S2.** **Details and amplification conditions of COI primers used in this study.**

| **Primer** | **Direction** | **Sequence (5’ to 3’)** | **Reference** | **PCR conditions** |
| --- | --- | --- | --- | --- |
| RepCOI | Foward | TNTTMTCAACNAACCACAAAGA | [12] | 94 (3'); 94 (40”), 50 (30”), 72 (1') x 35; 72 (7') |
|  | Reverse | ACTTCTGGRTGKCCAAARAATCA | [12] |  |
| RepCOI-SOK | Foward | TCAACNAACCACAAAGATATCGGC | This study | 94 (3'); 94 (40”), 50 (30”), 72 (1') x 40; 72 (7') |
|  | Reverse | CTGGRTGKCCAAARAATCAGAATAGG | This study |  |
| RepCOI-OBG | Foward | AACCACAAAGATATCGGCACCC | This study | 94 (3'); 94 (40”), 50 (30”), 72 (1') x 40; 72 (7') |
|  | Reverse | CCAAARAATCAGAATAGGTGTTGG | This study |  |
